# Regioselective C(sp^2^)-C(sp^3^) Coupling Mediated by Classical and Rollover Cyclometalation

**DOI:** 10.3390/molecules29030707

**Published:** 2024-02-03

**Authors:** Lorenzo Manca, Giacomo Senzacqua, Sergio Stoccoro, Antonio Zucca

**Affiliations:** 1 Dipartimento di Scienze Chimiche, Fisiche, Matematiche e Naturali, Università degli Studi di Sassari, Via Vienna 2, 07100 Sassari, Italy; lorenzmanc@tiscali.it (L.M.); giacomo.senzacqua@gmail.com (G.S.); stoccoro@uniss.it (S.S.); 2 Consorzio Interuniversitario Reattività Chimica e Catalisi (CIRCC), Villa La Rocca, Via Celso Ulpiani, 27, 70126 Bari, Italy

**Keywords:** cyclometalated complexes, C-H bond activation, Pt(II) complexes, rollover platinum complexes, nitrogen ligands

## Abstract

By taking advantage of a sequence of oxidative addition/reductive elimination reactions, Pt(II) cyclometalated derivatives are able to promote a rare C(sp^2^)-C(sp^3^) bond coupling, resulting in the production of novel methyl-substituted pyridines and bipyridines. Starting from 6-phenyl-2,2′-bipyridine, the step-by-step full sequence of reactions has been followed, leading to the unprecedented 3-methyl-6-phenyl-2,2′-bipyridine, which was isolated and fully characterized. The synthesis involves the following steps: (1) rollover cyclometalation to give the starting complex [Pt(N^C)(DMSO)Me]; (2) the synthesis of a more electron-rich complex [Pt(N^C)(PPh_3_)Me] by the substitution of DMSO with triphenylphosphine; (3) oxidative addition with methyl iodide to give the Pt(IV) complex [Pt(N^C)(PPh_3_)(Me)_2_(I)]; (4) iodide abstraction with silver tetrafluoborate to give an unstable pentacoordinate intermediate, which rapidly evolves through a carbon–carbon reductive coupling, forming a new C(sp^3^)-C(sp^2^) bond; (5) finally, the extrusion and characterization of the newly formed 3-methyl-6-phenyl-2,2′-bipyridine. The reaction has been therefore extended to a well-known classical cyclometalating ligand, 2-phenylpyridine, demonstrating that the method is not restricted to rollover derivatives. Following the same step-by-step procedure, 2-phenylpyridine was converted to 2-*o*-tolyl-pyridine, displaying the potential application of the method to the larger family of classical cyclometalated complexes. The application of this protocol may be useful to convert an array of heterocyclic compounds to their methyl- or alkyl-substituted analogs.

## 1. Introduction

**Scheme 1:**
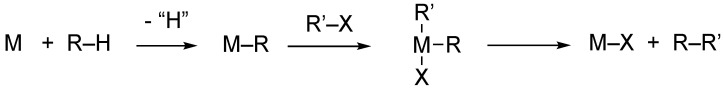
Activation, metalation, and functionalization via C-C reductive coupling.

One of the main goals of organometallic chemistry is the functionalization of C−H bonds mediated by transition metals, particularly when it involves the formation of new carbon–carbon bonds [1]. The C-C coupling is particularly interesting when it implicates the activation of a C-H bonds in organic compounds followed by metalation (the formation of a metal–carbon bond) and successive functionalization (Scheme 1):

A successive combination of oxidative addition/reductive elimination reactions is often found in catalytic cycles, leading first to the activation of a substrate and second to the formation of new bonds and hence to newly designed compounds. Reductive elimination is usually the key step in C−C bond formation, representing the reaction in which the final product is formed [2].

The cyclometalation reaction is a useful method for C-H bond activation in aromatic heterocycles, leading to particularly stable organometallic complexes known as cyclometalated compounds [3]. These derivatives, characterized by a carbon-heteroatom chelating ligand, have attracted enormous interest for various applications: as catalysts for a large array of reactions [4], in biomedicine as antitumor or antimicrobial agents [5,6], and in the field of innovative materials, such as photoluminescent devices, sensors, switches, etc. [7,8].

Furthermore, after the activation of the C-H bond, the subsequent functionalization of the cyclometalated ligand may facilitate the synthesis of new organic molecules by means of stoichiometric or catalytic processes [9].

**Figure 1:**
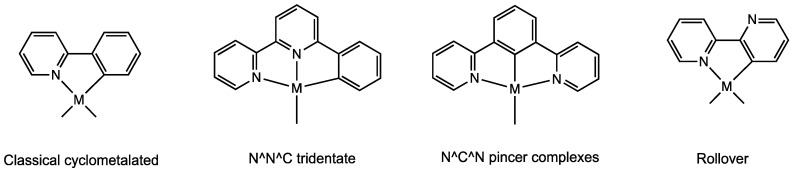
Some classes of cyclometalated complexes.

For these reasons, a great variety of subclasses of cyclometalated species have emerged, particularly for nitrogen donors such as pyridines, imines, and amines. In addition to classical C^N donors (e.g., phenylpyridines and phenylamines), the subclasses of tridentate cyclometalates have been thoroughly studied, comprising N^N^C donors [10] (such as 6-phenyl-2,2′-bipyridine) and the so-called “pincer ligands” [11] (e.g., C^N^C, N^C^N donors). One recent and unusual class is that of the rollover complexes, ref. [12] derived from the internal rotation of bidentate heterocyclic ligands, such as 2,2′-bipyridine (Figure 1).

Among the various methods, the rollover cyclometalation reaction is a quite new and useful process, suitable for activating heterocyclic donor ligands in remote positions, such as the C_3_ position in 2,2′-bipyridines (see Figure 1). It is not easy to functionalize these bidentate ligands in certain positions with transition metals owing to their propensity to form stable N^N chelated complexes. In contrast to classical cyclometalated complexes, rollover derivatives contain a free donor atom that confers behaviors not accessible to classical cyclometalated species [13], such as protonation [14], retro-rollover reactions [15,16], successive additional cyclometalation, and polymerization [17].

Rollover complexes were also found to promote C-C bond formation in both stoichiometric (gas phase and in solution) [18,19] and catalytic [20,21,22] reactions. In this regard, we have recently reported that the rollover activation of the C_3_-H bond in 2,2′-bipyridine Pt(II) rollover complexes might be employed to promote C-C bond formation by means of a sequence of oxidative addition and reductive elimination reactions to give methyl-substituted pyridine rings [23].

Following our previous studies in the field [24], we report some aspects of the behavior of one of the most studied organometallic ligands, 6-phenyl-2,2′-bipyridine (bpy^Ph^), which lead to the activation and functionalization of the remote pyridine C_3_-H bond through Pt(II) rollover cyclometalation. The reaction has been successfully extended to the corresponding classical ligand, 2-phenylpyridine (py^Ph^), revealing additional potential applications.

## 2. Results and Discussion

**Scheme 2:**
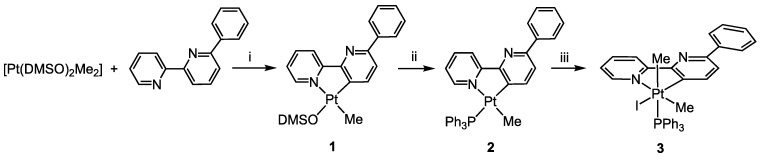
Synthesis of complex **3**: (i) acetone, 50 °C, 2 h; (ii) acetone, PPh_3_, r.t., 1 h; (iii) CH_2_Cl_2_, MeI, 30 °C, 6 h.

As reported previously, the reaction of the electron-rich Pt(II) precursor [Pt(DMSO)_2_(Me)_2_] with 6-phenyl-2,2′-bipyridine, bpy^Ph^, produces the rollover complex [Pt(bpy^Ph^-H)(Me)(DMSO], **1**, from which the substitution of the labile DMSO ligand allows the isolation of the corresponding phosphane complex [Pt(bpy^Ph^-H)(Me)(PPh_3_)], **2** [25]. The reaction can be performed following a “one-pot” procedure by adding PPh_3_ to the reaction mixture of [Pt(DMSO)_2_(Me)_2_] and bpy^Ph^ after completion of the rollover process, thus directly isolating **2** in high yield (Scheme 2).

Oxidative addition reactions of alkyl halides to Pt(II) electron-rich complexes have been extensively studied by several authors [26,27,28,29,30]. In particular, cyclometalated Pt(II) complexes are known to result in oxidative addition with methyl iodide, affording the corresponding Pt(IV) derivatives [31]. The reaction usually proceeds through an intermolecular S_N_2 mechanism at room temperature.

**Figure 2:**
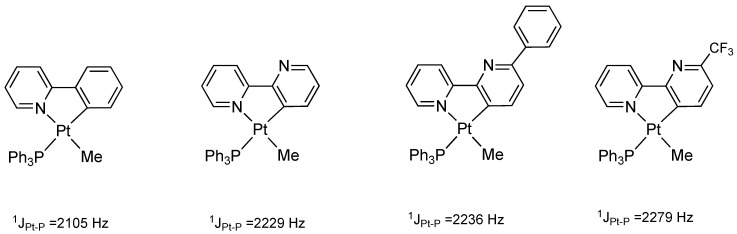
^195^Pt-^31^P NMR coupling constants in [Pt(N^C)(PPh_3_)Me] complexes as a consequence of N^C ligand donor properties [12,24,25].

The reaction of complex **2** with MeI does not occur easily: under the same conditions followed by analogous cyclometalated Pt(II) complexes (acetone, room temperature), we were not able to obtain the desired Pt(IV) product. The greater difficulty of the oxidative addition reaction on complex **2** may be explained by the donor properties of the rollover-coordinated 6-phenyl-2,2′-bipyridine. As we recently reported [32], a good indicator of the donor properties of platinum(II) cyclometalated complexes is the direct ^195^Pt-^31^P coupling value revealed by ^31^P NMR spectroscopy. In comparison with related cyclometalated ligands (Figure 2), 6-phenyl-2,2′-bipyridine is a poorer donor than cyclometalated 2-phenylpyridine and rollover-coordinated 2,2′-bipyridine, but better than the worst donor (6-CF_3_-2,2′-bipyridine). As a consequence, the platinum center in complex **2** is relatively electron-poor compared with similar cyclometalated complexes and the hampered oxidative addition reaction has a plausible justification.

It is likely that the presence of the phenyl substituent slows down the reaction, so we raised the temperature and prolonged the reaction time. In this way, we were able to obtain and isolate the Pt(IV) complex [Pt(bpy^Ph^-H)I(Me)_2_PPh_3_], **3**, in high yield (Scheme 2).

The reaction of **2** with MeI should give a complex with the methyl and iodide ligands entering in a mutually trans position. However, as previously observed, rapid isomerization produced the more stable isomer, with the phosphane ligand in the axial position with respect to the bipyridine metalated plane [33].

**Figure 3:**
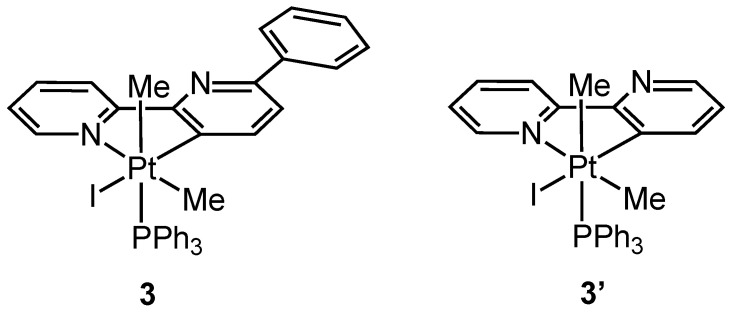
Pt(IV) derivatives **3** and **3**′.

Complex **3** was isolated and characterized analytically and spectroscopically, in particular, by means of NMR spectroscopy. The ^31^P NMR spectrum of **3** shows a singlet with satellites at −10.95 ppm, with a Pt-P coupling constant value, ^1^J_Pt-P_ = 962.5 Hz, in agreement with a Pt(IV) species, but smaller than that found in the corresponding simple 2,2′-bipyridine rollover complex **3′**, [Pt(bpy-H)(Me)_2_I(PPh_3_)] (^1^J_Pt-P_ = 991 Hz) (Figure 3).

The ^1^H NMR spectrum shows two different coordinated methyls as doublets with satellites due to coupling to ^195^Pt and ^31^P nuclei. Comparison with NMR data for **3′** shows that the shielded methyl (δ 1.24 ppm, ^2^J_Pt-H_ = 60.0 Hz) is coordinated in the “axial” position (with respect to the bipyridine plane), whereas the deshielded methyl (δ 1.70 ppm, ^2^J_Pt-H_ = 70.5 Hz) is in the equatorial position. The H_6′_ signal is strongly deshielded (δ 9.62 ppm, J_Pt-H_ *ca* 11 Hz), as expected, due to proximity to the iodide ligand [34,35].

### Treatment of Pt(IV) Complexes with Silver Salts

Compound **3** is stable in the presence of air and moisture, both in the solid state and in solution.

**Scheme 3:**
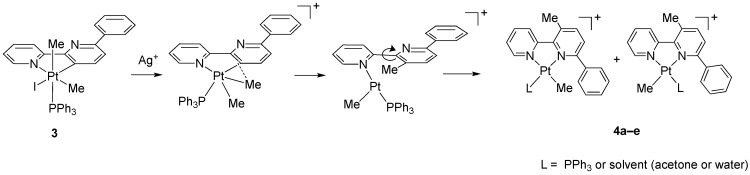
Reaction of **3** with AgBF_4_ (acetone, room temperature, 3 h).

The reaction of **3** with AgBF_4_ in acetone resulted in the abstraction of the coordinated iodide and the precipitation of solid AgI, which was removed by filtration. The resulting coordinatively unsaturated, five-coordinate species is unstable and rearranges following a reductive elimination process, through C(sp^3^)-C(sp^2^) coupling, and a subsequent “retro-rollover” process, involving the rotation of the substituted pyridine, allows the coordination of the second nitrogen atom to prepare N^N chelated adducts (Scheme 3).

**Figure 4:**
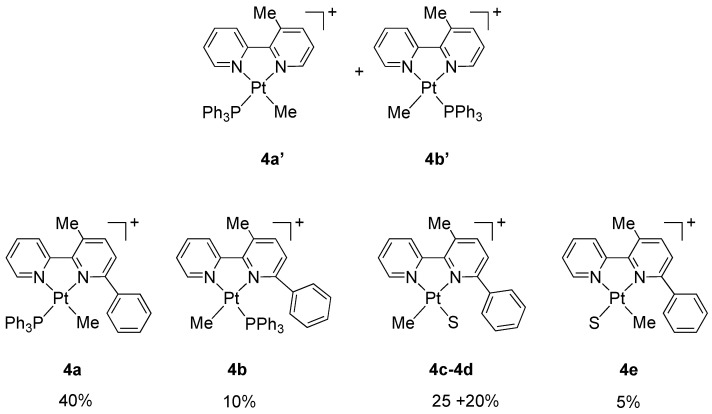
Adducts **4a′** and **4b′** obtained from 2,2′-bipyridine [23] and suggested formulations for species **4a**–**e** obtained from 6-phenyl-2,2′-bipyridine (this work). S = acetone or water, counter anion BF_4_^−^.

On the basis of our previous experience, we expected to obtain the two geometric isomers of the adduct [Pt(N^N)(PPh_3_)Me]^+^, as in the case of unsubstituted 2,2′-bipyridine, where the two species **4a′** and **4b′** were isolated in a 1:1 molar ratio (Figure 4). However, in the case of 6-phenyl-2,2′-bipyridine, likely due to the presence of the bulky phenyl substituent, the situation is more complex. The ^1^H NMR spectrum of the isolated product showed a mixture of at least five species in an approximate 8:5:4:2:1 molar ratio (Supplementary Material S3). All species contain a coordinated 3-methyl-6-phenyl-2,2′-bipyridine resulting from the desired C(sp^3^)−C(sp^2^) coupling, as shown by singlets around 3.0 ppm in a 1:1 molar ratio with the corresponding Pt-CH_3_ signals.

A tentative analysis of the complex mixture may explain the signals as follows: two species, present in a relative 4:1 molar ratio (*ca* 40% and 10% of the total) may be described as the geometric isomers of the adduct [Pt(N^N)(PPh_3_)Me]^+^ (N^N = 3-methyl-6-phenyl-2,2′-bipyridine, **4a** and **4b**). This interpretation is based on the presence of two coordinated methyls appearing as doublets with satellites, due to coupling with ^195^Pt and ^31^P nuclei: one of the signals, corresponding to the major species, is highly shielded (**4a**, *ca* 40%, δ −0.48 ppm, J_P-H_ = 3.8 Hz, J_Pt-H_ = 69.5 Hz) as it is in proximity of the shielding cone of the phenyl substituent of the bipyridine, whereas the other signal (**4b**, *ca* 10%, δ 0.86 ppm, J_P-H_ = 3.6 Hz, J_Pt-H_ = *ca* 70 Hz) appears in the usual region at around 0.9–1.1 ppm. Two singlets, at 2.99 and 3.07 ppm, with the same integral values and 4:1 molar ratio, may be assigned to the methyls on the C_3_ position in the central pyridine ring. In the ^31^P NMR spectrum, these species gave singlets with satellites at 17.57 ppm (**4a**, J_Pt-P_ = 4581 Hz) and 12.49 ppm (**4b**, J_Pt-P_ = 4397 Hz), with the same 4:1 molar ratio. Other smaller signals in the ^31^P NMR spectrum, e.g., a singlet at 31.72 ppm, were not interpreted.

In the ^1^H NMR spectrum, two other compounds, **4c** and **4d**, in an approximate 5:4 molar ratio, (ca. 25% and 20% of the total, respectively), may tentatively be described as solvato species [Pt(N^N)(S)Me]^+^ (S = acetone or water) generated from detachment of the PPh_3_ ligand; these species show two singlets with satellites, indicating the absence of coordinated PPh_3_ (**4c**, 25%: δ 1.04 ppm, J_Pt-H_ = 71.5 Hz; **4d**, 20%: δ 0.96 ppm, J_Pt-H_ = 67.5 Hz). Two singlets at 2.63 and 2.88 ppm (**4c** and **4d** respectively, Me on C_3_) indicates that also these species are derived from the C-C coupling. Furthermore, two signals at 3.80 and 2.19 ppm (the latter overlapping with free acetone) may support the assignment of **4c** and **4d** as solvato species. Finally, a small singlet with satellites, at −0.17 ppm (ca. 5%, J_Pt-H_ = 66.5 Hz), may be a solvato species (**4e**) with a methyl coordinated close to the phenyl substituent.

It is worth mentioning that C(sp^2^)-C(sp^3^) formation by reductive elimination is relatively rare, both catalytically and stoichiometrically [36,37,38,39,40,41,42,43]. In our previous study [23] of 2,2′-bipyridine, 2-pyridylquinoline, and 6-methyl-2,2′-bipyridine, we tried to determine which of the two methyl ligands coordinated to platinum(IV) was involved in the coupling. However, the oxidative addition reaction with deuterated CD_3_I produced a 50:50 distribution of CH_3_ and CD_3_ coordinated in the axial and equatorial positions, explainable by a rapid isomerization of the pentacoordinated Pt(IV) complex [Pt(N^C)(CD_3_)(CH_3_)(PPh_3_)]^+^ before the coordination of iodide.

Here, our main interest consisted in the isolation of the new 3-methyl-6-phenyl-2,2′-bipyridine, so we did not devote further efforts to the characterization of species **4a**–**e**, and proceeded with the decomplexation of the ligand from the metal.

The free-substituted 2,2′-bipyridine **5** was isolated by reaction of the **4a**–**e** species with dppe (1,2-bis-diphenylphosphinoethane). The chelating diphosphane easily displaces the bipyridine ligand so that the organic species can be isolated in good yield (ca. 80%) and analyzed.

To the best of our knowledge, compound **5** has not previously appeared in the literature and its synthesis is unprecedented. For this reason, we performed a thorough characterization in solution by means of mono- and two-dimensional ^1^H and ^13^C NMR spectroscopy (^1^H, ^13^C, H-H COSY, H-H NOESY, H-C HMQC, H-C HMBC).

The ^1^H and ^13^C NMR spectra clearly show the methyl in position 3 (^1^H, 2.58 ppm, ^13^C, 19.98 ppm). The aromatic region shows all the expected hydrogens, with 4, 2, and 5-proton spin systems for the aromatic rings, for which a COSY H-H spectrum allowed the assignment of all ^1^H NMR signals.

**Figure 5:**
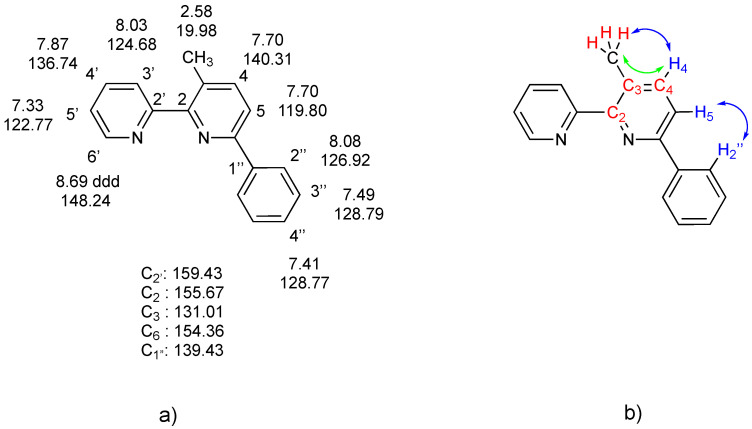
(**a**) (left): 3-Methyl-6-phenyl-2,2-bipyridine, **5**, along with numbering scheme and complete ^1^H and ^13^C NMR assignment (CDCl_3_ solution) deduced from 2D NMR spectra; (**b**) (right) interactions revealed by 2D NMR spectra: (i) in red, proximity of CH_3_ protons with C_2_, C_3_, and C_4_, shown by cross peaks in the HMBC spectrum; (ii) in blue, NOE contacts between CH_3_ and H_2”_ with the H_4_/H_5_ spin system (NOESY spectrum); (iii) in green, long-range interactions of CH_3_ protons and H_5_, revealed by the COSY spectrum.

In addition, ^1^H-^13^C HSQC and HMBC spectra allowed the assignment of ^13^C signals. A complete assignment is reported in Figure 5.

In particular, the 2D ^1^H-^13^C HMBC NMR spectrum provided fundamental information, revealing intense cross peaks for ^3^J(C-H) coupling, as well as weak peaks for ^2^J(C-H) and ^4^J(C-H) couplings. As an example, the ^1^H methyl singlet at 2.58 ppm shows cross peaks with signals at 155.52 (C_2_), 130.9 (C_3_), and 140.16 (C_4_) ppm, supporting the given formulation. The analysis of the other peaks allowed complete assignment. The bonding of the methyl group on the C_3_ position is confirmed by the 2D COSY spectrum, which shows a weak long-range interaction between the methyl protons at 2.58 ppm and the H_4_ signal at 7.70 ppm (part of an AB system with the H_5_ hydrogen).

The ^1^H NMR spectrum of **5** in CD_2_Cl_2_ slightly separated the H_4_ and H_5_ signals in the AB system (δ 7.73, 7.75 ppm, J_AB_ = 8.1 Hz). A NOESY spectrum showed NOE contacts between the AB system and the methyl at 2.60 ppm, as well as with the H_2”_ protons; a detailed analysis, hampered by the tightness of the AB system, allowed assignment of the H_4_ and H_5_ signals (δ 7.73 and 7.75 ppm, respectively).

The GC-MS spectrum (EI) of **5** shows peaks at *m*/*z* 245.2 (100%, [M-H]^+^) and 246.2 ([M]^+^, calculated for C_17_H_14_N_2_ 246.12).

**Scheme 4:**
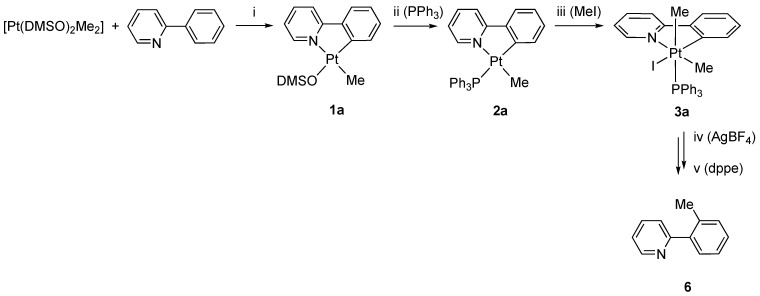
Synthesis of **6**: (i) toluene, 110 °C, 3 h; (ii) toluene, PPh_3_, r.t., 1 h; (iii) CH_2_Cl_2_, MeI, 30 °C, 6 h; (iv) CH_2_Cl_2_/acetone 5:1, AgBF_4_, 3 h, r.t.; (v) CH_2_Cl_2_/acetone 5:1, dppe, 15′.

Functionalization in the C_3_ position of 2,2′-bipyridines is not so common: the methods reported in literature comprise Negishi [44] and Suzuki–Miyaura coupling [45], in addition to co-cyclotrimerization reactions [46]. In our previous investigation, we reported that only rollover complexes were able to follow the sequence of reactions also reported in this paper. The intermediate adducts [Pt(N^N)(PPh_3_)Me]^+^, analogous to **4a**–**e**, were stabilized by chelation, a behavior not available for classical ligands such as 2-phenylpyridine. However, the retro-rollover process should follow the C-C coupling, so we analyzed in more detail the reaction starting from the classical analog [Pt(N^C)Me(PPh_3_)] complex derived from 2-phenylpyridine, **6** (Scheme 4).

Following the same reaction sequence used for **1**–**4**, we obtained the Pt(IV) species **3a**, which was isolated in the solid state and analyzed by means of NMR spectroscopy.

In this case, we did not try to isolate adduct species analogous to **4a**–**e**, as we were mainly interested in the isolation of the final organic compound. Owing to the different solubility of **3a** with respect to **3**, the reaction with silver tetrafluoborate was performed in a CH_2_Cl_2_/acetone 5/1 molar ratio mixture. Analogous to the previous case, the addition of AgBF_4_ followed by displacement with dppe, 1,2-bis(diphenylphosphine)ethane, led to the isolation of 2-*ortho*-tolylpyridine, **6**, derived from the C(sp^3^)-C(sp^2^) coupling. Compound **6** was purified by acid–base extraction and characterized by NMR spectroscopy. The spectroscopic data, in agreement with the previously reported data for **6**, ref. [47] revealed, *inter alia*, the presence of the methyl group at 2.41 and 20.26 ppm, in the ^1^H and ^13^C spectra, respectively.

Furthermore, the GC-MS spectrum (EI) shows peaks at *m*/*z* 168.1 (100%, [M-H]^+^) and 169.1 ([M]^+^, calculated for C_12_H_11_N 169.09).

The obtained result is significant because, to the best of our knowledge, this is a new strategy for C-C coupling in a classical cyclometalated complex, following this sequence of reactions. Moreover, this result suggests the possibility of extending the functionalization to a wide range of cyclometalated ligands.

## 3. Experimental Section

All the solvents were purified and dried according to standard procedures [48]. *Cis*-[Pt(DMSO)_2_(Me)_2_] was synthesized according to reference [49,50]. Elemental analyses were performed with a PerkinElmer elemental analyzer 240B.^1^H, ^13^C{^1^H}, and ^31^P{^1^H} NMR spectra were recorded using a Bruker Avance III 400 spectrometer operating at 400.132, 100.623 and 161.967 MHz, respectively. Chemical shifts are given in ppm relative to internal TMS for ^1^H and ^13^C{^1^H} and external 85% H_3_PO_4_ for ^31^P{^1^H}; J values are given in Hz. ^1^H-^1^H COSY, ^1^H-^1^H NOESY, ^1^H-^13^C HSQC, and ^1^H-^13^C HMBC experiments were performed by means of standard pulse sequences.

GC-MS analyses were carried out using an Agilent GC 7820A equipped with an Agilent MSD 5977E (Column: Zebron ZB-5 60 m × 0.25 mm, i.d. × 0.25 µm).

### Preparations

Synthesis of **2**, [Pt(bpy^Ph^-H)Me(PPh_3_)]

Complex **2** was obtained as previously reported [20], following a one-pot procedure, adding PPh_3_ after completion of the reaction between *cis*-[Pt(DMSO)_2_(Me)_2_] and 6-phenyl-2,2′-bipyridine to give complex **1** as an intermediate. Yield 95%. ^1^H NMR: (400.1 MHz, CDCl_3_): δ (ppm) 8.55 (d, 1H, H_6′_), 8.33 (dd with sat, ^3^J_Pt-H_ = 47.1 Hz), 8.20–7.38 (m, aromatics), 6.71 (m, 1H), 0.79 (d with sat, 3H, ^2^J_Pt-H_ = 83.2 Hz, CH_3_). ^31^P NMR: (136.0 MHz, CDCl_3_): δ (ppm) 32.49 (s with sat, ^1^J_Pt-P_ = 2236 Hz, PPh_3_).

Synthesis of **3**, [Pt(bpy^Ph^-H)I(Me)_2_PPh_3_]

To a solution of [Pt(bpy^Ph^-H)Me(PPh_3_)] (300.0 mg; 0.42 mmol) in 15 mL of dichloromethane, methyl iodide (159.0 μL; 2.52 mmol) was added under stirring. The solution was heated to 30 °C for 6 h, under an argon atmosphere. After completion, the solution was concentrated to a small volume and treated with hexane. The precipitate formed was filtered off and dried to give the analytical sample (331.0 mg; Yield: 93%). Elemental analysis: calculated for C_36_H_32_IN_2_PPt. (%): C, 51.13; H, 3.81; N, 3.31; found: C, 51.26; H, 3.92; N, 3.19. ^1^H NMR: (400.1 MHz, CDCl_3_): δ (ppm) 9.65 (dd with sat, 1H, ^2^J_H-H_ = 5.6 Hz J_Pt-H_ = 11.5 Hz, H6′); 8.42 (d, 1H), 8.07 (m, 2H), 7.80 (td, 1H), 7.57–7.05 (m, 21H + CDCl_3_), 7.06 (dd, 1H), 1.64 (d with sat, 3H, ^2^J_P-H_ = 7.7 Hz,^2^J_Pt-H_ = 70.45 Hz, CH_3_). 1.17 (d with sat, 3H, ^2^J_P-H_ = 7.4 Hz, ^2^J_Pt-H_ = 60.0 Hz, CH_3_).^31^P NMR: (136.0 MHz, CDCl_3_): δ (ppm) −10.9 (s with sat, ^1^J_Pt-P_ = 962.5 Hz, PPh_3_).

Reaction of **3** with AgBF_4_

To a solution of **3** (255.3 mg; 0.30 mmol) in acetone (20 mL) AgBF_4_ (61.4 mg; 0.30 mmol) was added under stirring at room temperature in an argon atmosphere. After three hours, the formed AgI was removed by filtration over celite. The filtered solution was concentrated to a small volume and treated with a hexane–diethyl ether 1:5 mixture to give a precipitate that was filtered off and dried to give the analytical sample.

The ^1^H NMR spectrum of the obtained product shows the presence of at least five species, **4a** (40%), **4b** (10%), **4c** (25%), **4d** (20%), **4e** (5%), tentatively identified as [Pt(N^N)L(Me)]^+^ cationic adducts (L = PPh_3_, water, or acetone) where the N^N ligand is 3-methyl-6-phenyl-2,2′-bipyridine (**5**).

Selected ^1^H and ^31^P NMR data (CDCl_3_, δ (ppm))

**4a** (40%), [Pt(Pt(k^2^-N,N-3-methyl-6-phenyl-2,2′-bipyridine)(PPh_3_)(Me)]^+^ (isomer with Pt-Me *cis* to Ph-bipy): ^1^H NMR: δ −0.47 (d with sat, 3H, J_P-H_ = 3.8 Hz, J_Pt-H_ = 69.5 Hz, Pt-Me); 2.99 (s, 3H, Me-bipy) ^31^P NMR: δ 17.57 (s with sat, J_Pt-P_ = 4581 Hz).

**4b** (10%), [Pt(Pt(k^2^-N,N-3-methyl-6-phenyl-2,2′-bipyridine)(PPh_3_)(Me)]^+^ (isomer with Pt-Me *trans* to Ph-bipy): δ 0.86 (d with sat, 3H, J_P-H_ = 3.6 Hz, J_Pt-H_ = ca 70 Hz, Pt-Me); 3.07 (s, 3H, Me-bipy). ^31^P NMR: δ 12.49 (s with sat, J_Pt-P_ = 4397 Hz).

**4c** (25%), [Pt(Pt(k^2^-N,N-3-methyl-6-phenyl-2,2′-bipyridine)(S)(Me)]^+^ (S = water or acetone, isomer with Pt-Me *trans* to Ph-bipy). ^1^H NMR: δ 1.04 (s with sat, 3H, J_Pt-H_ = 71.5 Hz, Pt-Me); 2.63 (s, 3H, Me-bipy).

**4d** (20%), [Pt(Pt(k^2^-N,N-3-methyl-6-phenyl-2,2′-bipyridine)(S)(Me)]^+^ (S = water or acetone, isomer with Pt-Me *trans* to Ph-bipy). ^1^H NMR: δ 0.96 (s with sat, 3H, J_Pt-H_ = 67.5 Hz, Pt-Me); 2.88 (s, 3H, Me-bipy).

**4e** (5%), [Pt(Pt(k^2^-N,N-3-methyl-6-phenyl-2,2′-bipyridine)(S)(Me)]^+^ (S = water or acetone, isomer with Pt-Me *trans* to Ph-bipy), ^1^H NMR: δ -0.15 (s with sat, J_Pt-H_ = 66.5 Hz, Pt-Me); 2.85 (s, 3H, Me-bipy).

Synthesis of **5**, 3-methyl-6-phenyl-2,2′-bipyridine

To a solution of **3** (255.3 mg; 0.30 mmol) in acetone (20 mL), AgBF_4_ (61.4 mg; 0.30 mmol) was added under stirring at room temperature in an argon atmosphere. After three hours, the formed AgI was removed by filtration over celite. To the filtered solution, a 10% excess of dppe (132.1 mg; 0.303 mmol) was added and the solution was left under stirring for 15′. Then the mixture was evaporated to dryness. The organic product was solubilized with diethyl ether, filtered and purified by an acid–base extraction in order to eliminate traces of dppe, anhydrified with Na_2_SO_4_, filtered and evaporated to dryness to give the analytical sample with a 75% yield. Complete ^1^H and ^13^C NMR assignment was enabled by analysis of 1D and 2D NMR spectra (^1^H, ^13^C, H-H COSY, H-H NOESY, H-C HSQC, H-C HMBC).

^1^H NMR: (400.1 MHz, CDCl_3_): δ (ppm) 8.69 (ddd, 1H, J_H-H_ = 4.8, 1.7, 0.9 Hz, H_6′_), 8.08 (m, 1H, H_2″_), 8.03 (dt, 1H, J_H-H_ = 8.0, 0.9 Hz, H_3′_), 7.87 (td, 1H, J_H-H_ = 7.8, 1.8 Hz H_4′_), 7.70 (AB system, 2H, H_5_+H_4,_ J_H5-H4_ = 8.1 Hz), 7.49 (m, 2H, H_3″_), 7.41 (m, 1H, H_4″_), 7.33 (ddd, 1H, J_H-H_ = 7.7, 4.8, 1.2 Hz, H_5′_), 2.58 (s, 3H, CH_3_). ^1^H NMR (400.1 MHz, CD_2_Cl_2_): selected signals δ (ppm) 8.71 (ddd, 1H, H_6′_), 7.73(H_4_, 1H, AB system, J_AB_ = 8.1 Hz); 7.75 (H_5_, 1H, AB system, J_AB_ = 8.1 Hz); 2.60 (s, 3H, CH_3_).

^13^C NMR: (100.1 MHz, CDCl_3_) δ (ppm) 159.43 (C_2_); 155.67 (C_2′_); 154.36 (C_6_); 148.24 (C_6′_); 140.31 (C_4_); 139.43 (C_1″_); 136.74 (C_4′_); 131.01 (C_3_); 128.79 (C_3″_); 128.77 (C_4″_); 126.92 (C_2″_); 124.68 (C_3′_); 122.77 (C_5′_); 119.80 (C_5_); 19.98 (CH_3_).

GC-MS (EI): C_17_H_14_N_2_, found *m*/*z* 245.2 [M-H]^+^ (calculated for M^+^ 246.1).

Synthesis of **2a**, [Pt(py^Ph^-H)Me(PPh_3_)]

To a solution of cis-[Pt(DMSO)_2_(Me)_2_] (210.0 mg; 0.55 mmol) in 15 mL of anhydrous toluene, 0.10 mL of 2-phenylpyridine (0.70 mmol) was added under constant stirring. The solution was heated, under an argon atmosphere, to a temperature of 110 °C for 3 h. After cooling to room temperature, a 10% excess of triphenylphosphine was added and allowed to react for one hour. The obtained solution was concentrated to a small volume and treated with diethyl ether. The precipitate formed was filtered off and washed with hexane to produce the analytical sample (0.174 g; yield: 50%). ^1^H and ^31^P NMR data match those previously reported [51].

Selected ^1^H NMR data: (400.1 MHz, CDCl_3_): δ (ppm) 077 (d with sat, 3H, ^3^J_P-H_ = 7.9 Hz, ^2^J_Pt-H_ = 83.5 Hz, Pt-Me). 8.00 (m with sat, 1H, J_Pt-H_ = 51 Hz, H_3′_), ^31^P NMR (136.0 MHz, CDCl_3_): δ (ppm) 33.10 (s with sat, J_Pt-P_ = 2105 Hz).

Synthesis of **3a**, [Pt(py^Ph^-H)I(Me)_2_(PPh_3_)]

To a solution of [Pt(py^Ph^-H)Me(PPh_3_)] (100.0 mg; 0.16 mmol) in 15 mL of dichloromethane, an excess of CH_3_I (59.6 μL; 0.96 mmol) was added, under constant stirring in an argon atmosphere. The solution was heated to 30 °C for 6 h. After completion, the solution was concentrated to small volume and treated with hexane. The precipitated formed was filtered off and dried to give the analytical sample (0.083 g; yield: 68%). ^1^H and ^31^P NMR data match those previously reported. Ref. [33] Selected NMR data (CDCl_3_): δ(ppm) 1.25 (d, 3H, ^2^J_Pt-H_ = 60.8 Hz, ^3^J_P-H_ = 7.5 Hz, Me *trans* to P), 1.65 (d, 3H, ^2^J_PtH_ = 71.1 Hz, ^3^J_P-H_ = 7.7 Hz, Me *trans* to N), 9.72 (ddd, 1H, J_H-H_) 5.5 Hz, ^3^J_Pt-H_ = 10.5 Hz, H_6′_, adjacent to N). ^31^P NMR (136.0 MHz, CDCl_3_): −9.34 (s, ^1^J_Pt-P_ = 967 Hz).

Synthesis of **6**, 2(*o*-tolyl)pyridine

To a solution of [Pt(py^Ph^-H)I(Me)_2_(PPh_3_)] (60.0 mg; 0.080 mmol) in a 5:1 mixture of dichloromethane acetone, AgBF_4_ (17.2 mg, 0.088 mmol) was added under constant stirring. The solution was stirred at room temperature, under an argon atmosphere, for three hours. At the end, the resulting suspension was filtered to remove the formed AgI and a 10% excess of 10% dppe (35.06 mg; 0.088 mmol) is added. The solution was stirred at room temperature for 15′, then evaporated to dryness, solubilized with diethyl ether, and filtered. The filtered solution was evaporated to dryness and purified by means of an acid–base extraction, followed by anhydrification with Na_2_SO_4_, filtration, and evaporation to dryness, to produce the analytical sample (0.015 g; yield: 94%). Selected NMR data. ^1^H NMR: (400.1 MHz, CDCl_3_): δ (ppm) 8.72 (dd, 1H, H_6′_), 7.81 (td, 1H, H_4′_), 2.41 (s, 3H, CH_3_).^13^C NMR: (100.1 MHz, CDCl_3_) δ (ppm) 159.83 (Cq); 148.91; 139.98 (Cq); 136.78, 135.92, 130.93, 129.80, 128.63, 126.07, 124.47, 121.93, 20.42 (CH_3_). NMR data match those previously reported (ref. [47]).

GC-MS (EI): C_12_H_11_N, found *m*/*z* 168.1 [M-H]^+^ (calculated for M^+^ 169.1). Traces (<1%) of triphenylphosphine oxide (*m*/*z* 278.1).

## 4. Conclusions

The coupling reaction reported here constitutes a new synthetic method for the preparation of methyl-substituted heterocycles: in principle, a large array of heterocyclic donors, able to support classical or rollover cyclometalation may follow the sequence of reactions reported here, taking advantage of a rollover/retro-rollover reaction pathway.

The reductive elimination step involves a rather rare C(sp^3^)-C(sp^2^) coupling in place of the more common C(sp^3^)-C(sp^3^) one. The reaction has now been demonstrated not to be restricted to rollover complexes but may have a more general application.

The next steps in this research will be the extension of the reaction to other cyclometalated heterocyclic donors and to other organic halides in order to add different groups in the C-C coupling. It will also be useful for revealing the factors governing the process, such as the electronic and steric influence of the ligands coordinated to the platinum center.

## Data Availability

Data available from the corresponding author on request.

## Supplementary Materials

The following are available online at https://www.mdpi.com/article/10.3390/molecules29030707/s1. NMR spectra of compounds **2**, **3**, **4**, **5**, **2a**, **3a**, **4a** and **6**: S1–S13; GC-MS spectra of **5** and **6**: S14 and S15.
