# Supplementary material for: Regioselective C(sp2)-C(sp3) Coupling Mediated by Classical and Rollover Cyclometalation
Source: Molecules. 2024 Feb 3;29(3):707. doi: 10.3390/molecules29030707 (PMC10856536; doi:10.3390/molecules29030707)

Supplementary material

Regioselective C(sp^2)-C(sp^3) coupling mediated by classical and rollover cyclometalation

Lorenzo Manca,[1] Giacomo Senzacqua,[1,2] Sergio Stoccoro [1,2] and Antonio Zucca [1,2,*]

1 Dipartimento di Scienze Chimiche, Fisiche, Matematiche e Naturali, Università degli Studi di Sassari, Via Vienna 2, 07100 Sassari, Italy
2 Consorzio Interuniversitario Reattività Chimica e Catalisi (CIRCC), Villa La Rocca, Via Celso Ulpiani, 27, 70126 Bari, Italy
* Correspondence: zucca@uniss.it

Contents

1) ^{1}H, ^{13}C{^{1}H} and ^{31}P{^{1}H} NMR spectra of compounds **2**, **3**, **4**, **5**, **2a**, **3a**, **4a** and **6**: S1-S13

2) GS-MS spectra of compounds **5** and **6**: S14-S15

^{1}H NMR spectrum of complex **2** ($CDCl_3$)

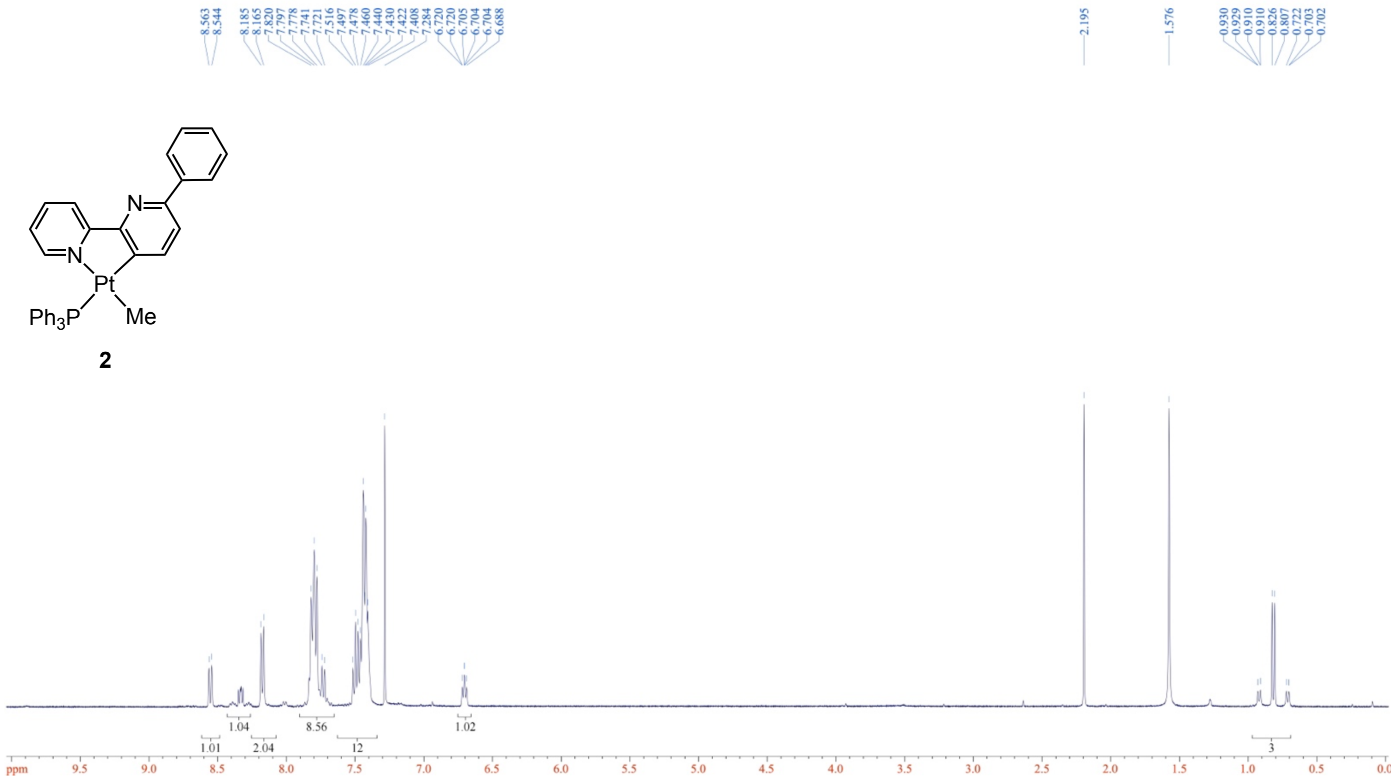

^{31}P NMR spectrum of complex **2** ($CDCl_3$)

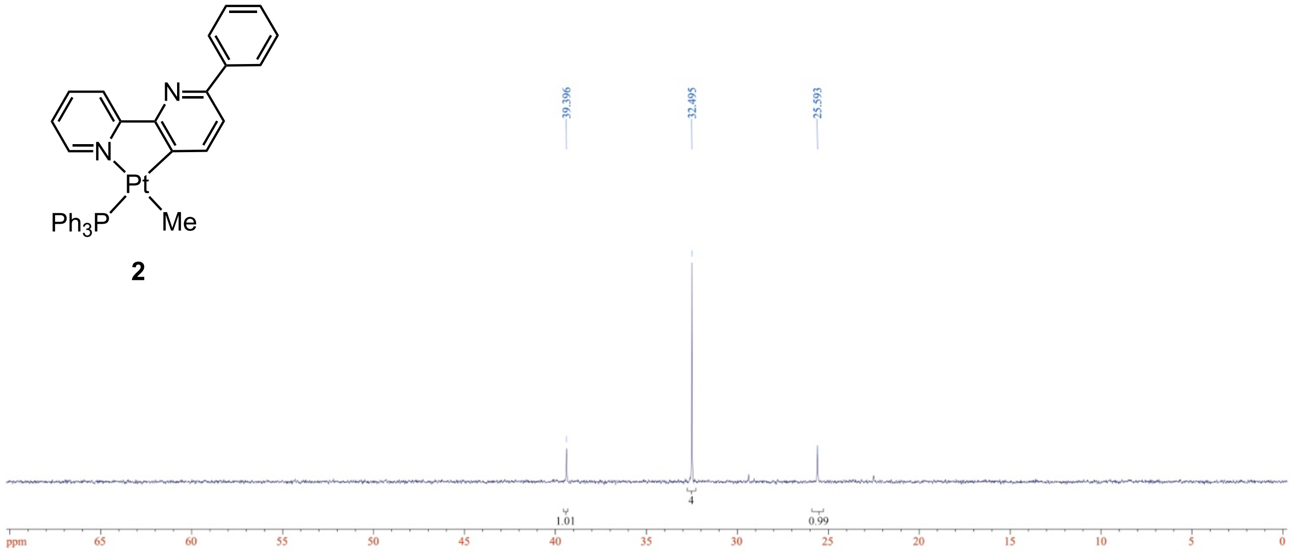

^{1}H NMR spectrum of complex **3** ($CDCl_3$)

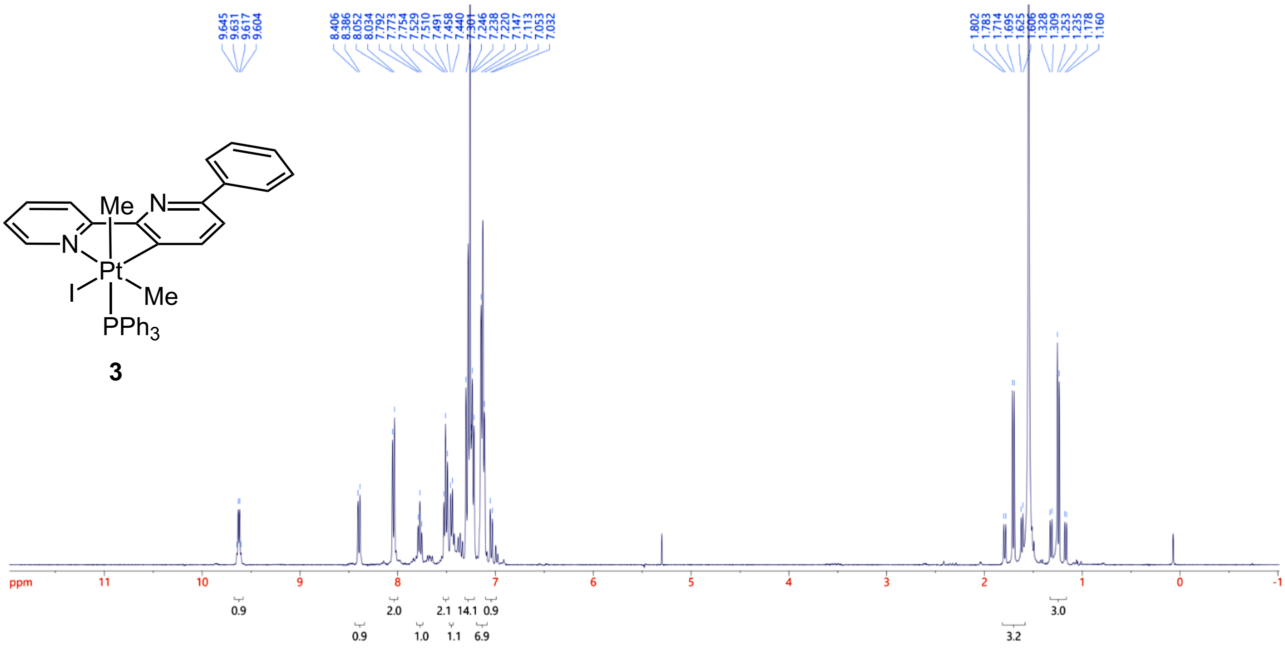

^{31}P NMR spectrum of complex **3** ($CDCl_3$)

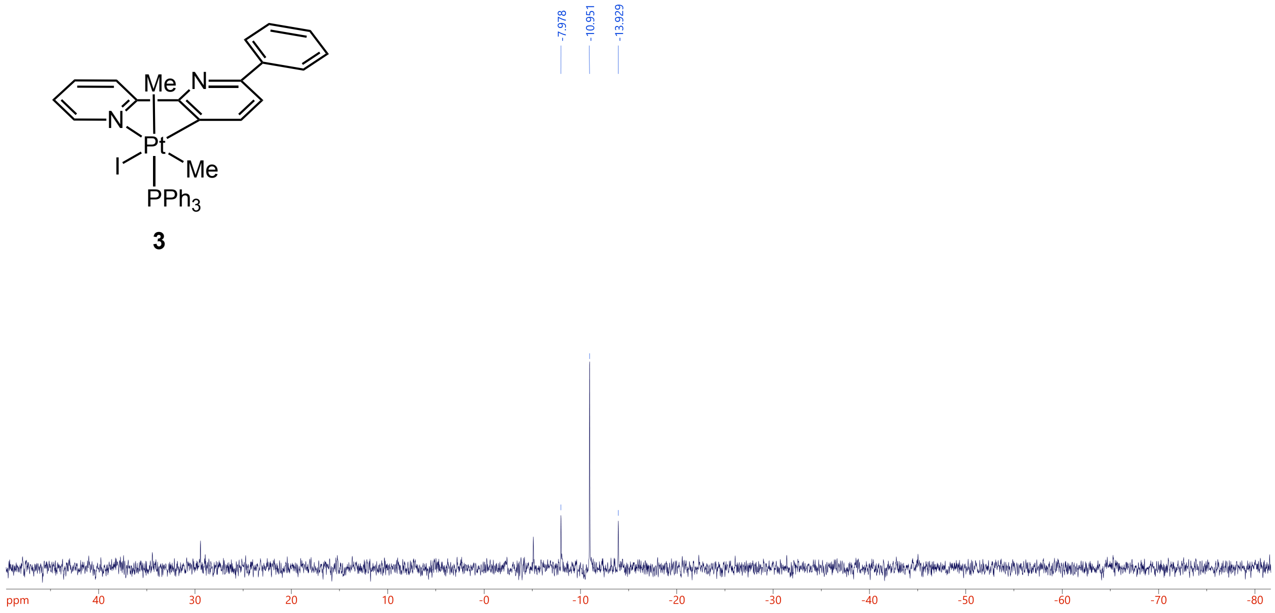

^{1}H NMR of complexes **4a-e** ($CDCl_3$)

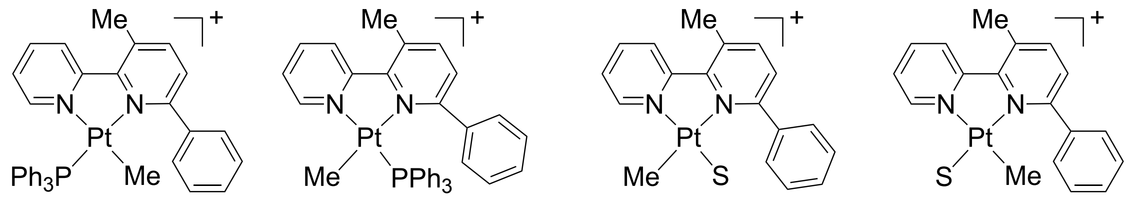

**4a**	**4b**	**4c-4d**	**4e**
40%	10%	25 +20%	5%

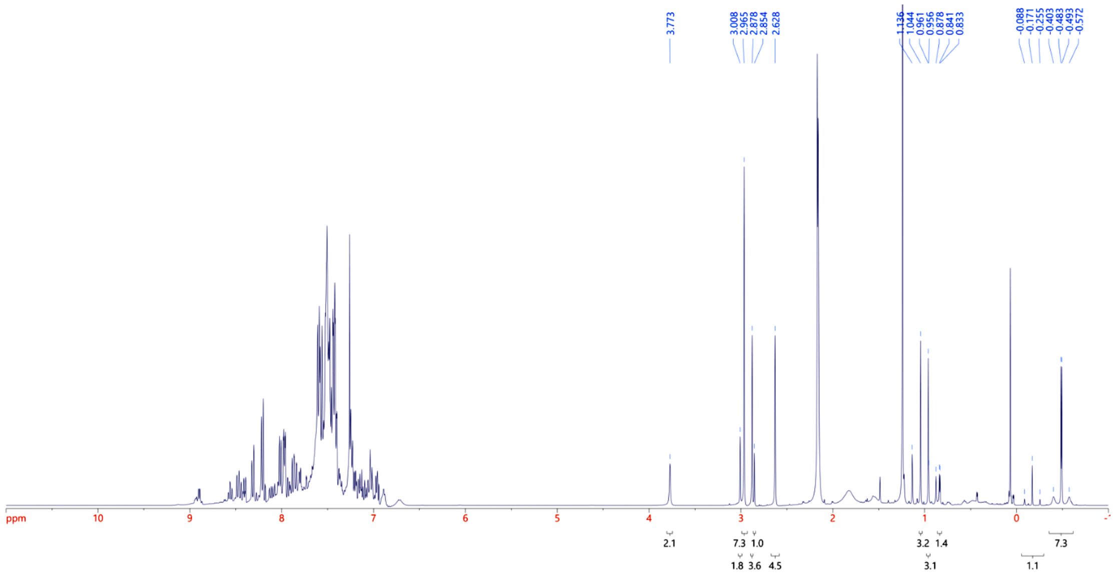

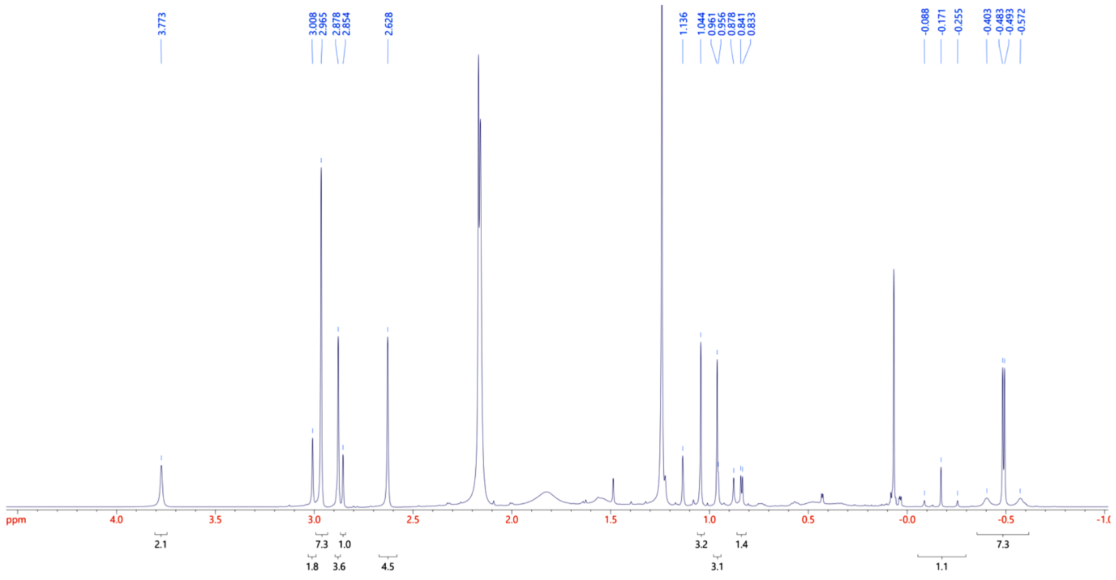

^{1}H NMR spectrum ($CDCl_3$, aliphatic region) of complexes **4a-e**

note: the signal at 0.96 ppm (integral = 3.1) overlaps a satellite of the signal at 1.04 ppm. For this reason only the central singlet has been integrated for these methyls. The corrected molar ratio of the signals at 1.04 ppm and 0.96 ppm is approx. 5:4, more or less the same than the two signals at 2.63 and 2.88 ppm. Also for the doublet with satellites at 0.83 ppm only the central doublet has been integrated.

^{31}P NMR of complexes **4a-e** ($CDCl_3$)

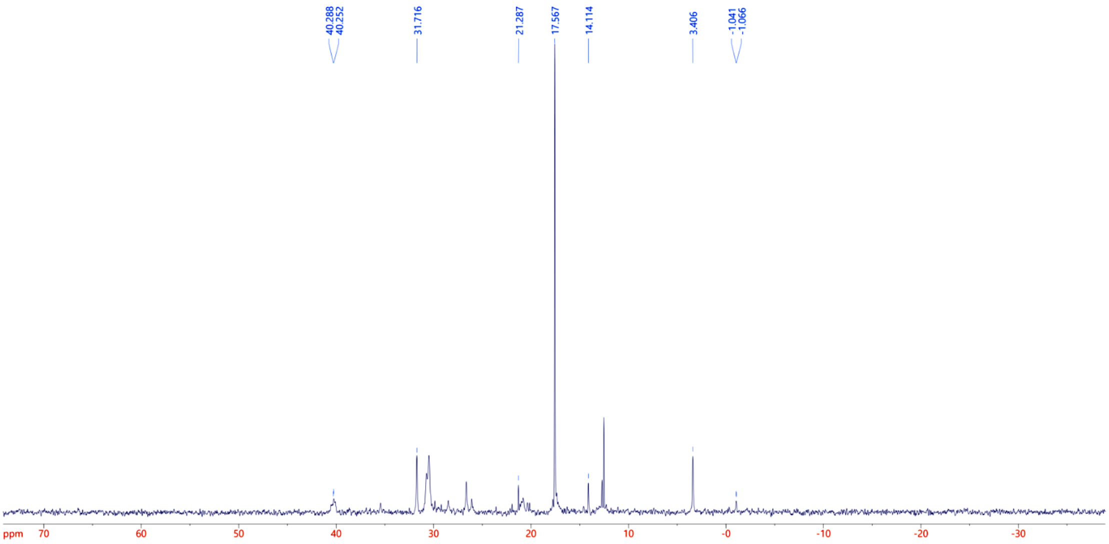

^{1}H NMR spectrum of compound **5** ($CDCl_3$)

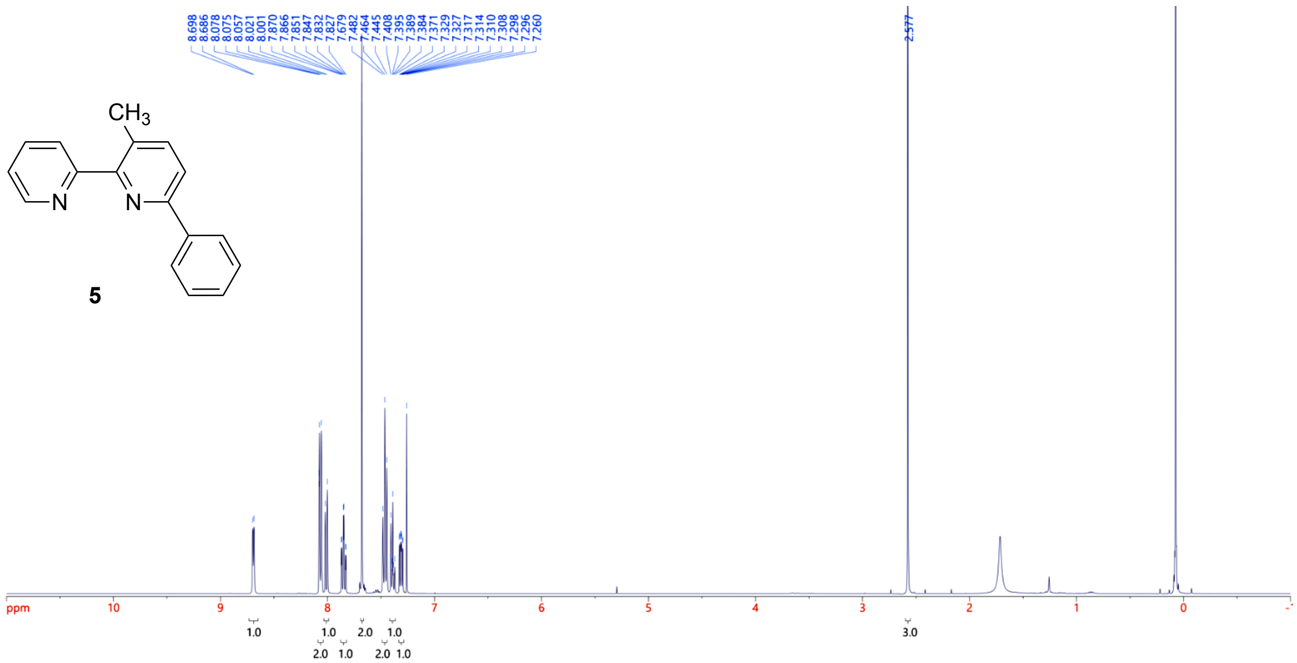

^{1}H NMR spectrum of compound **5** (aromatic region, $CDCl_3$)

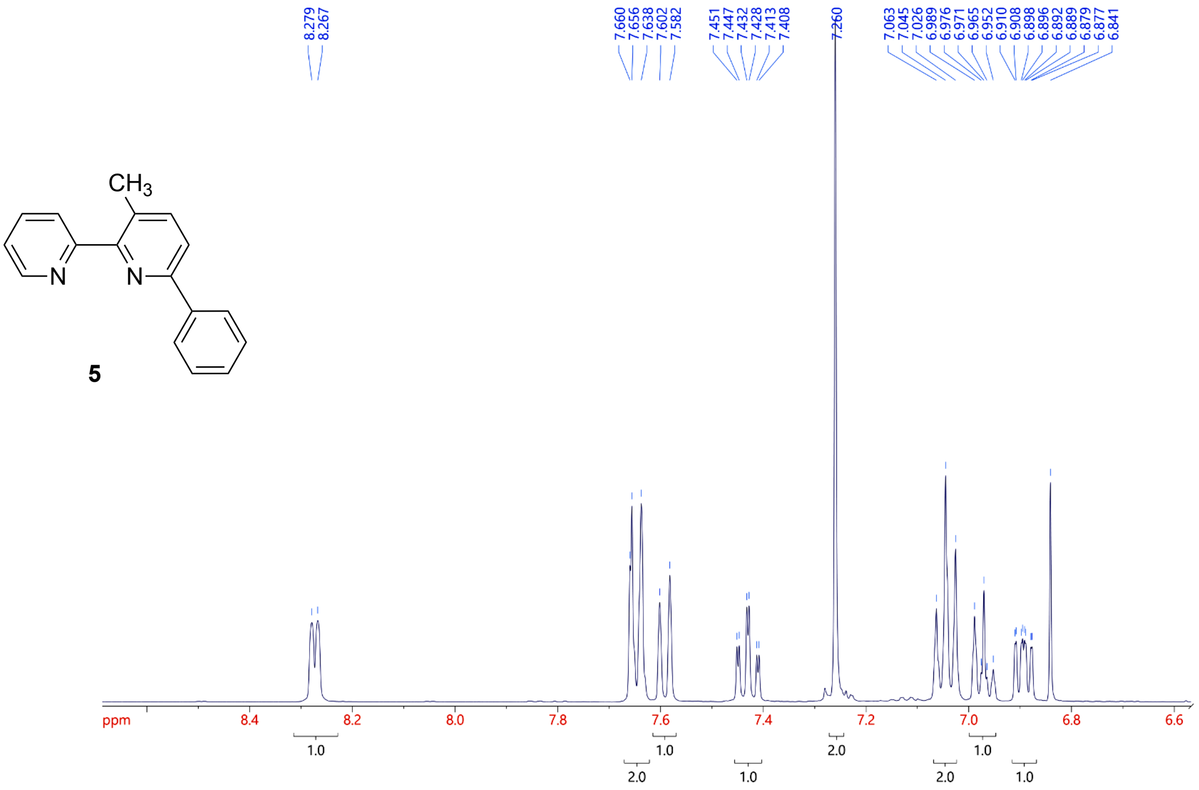

^{13}C NMR spectrum of compound **5** ($CDCl_3$)

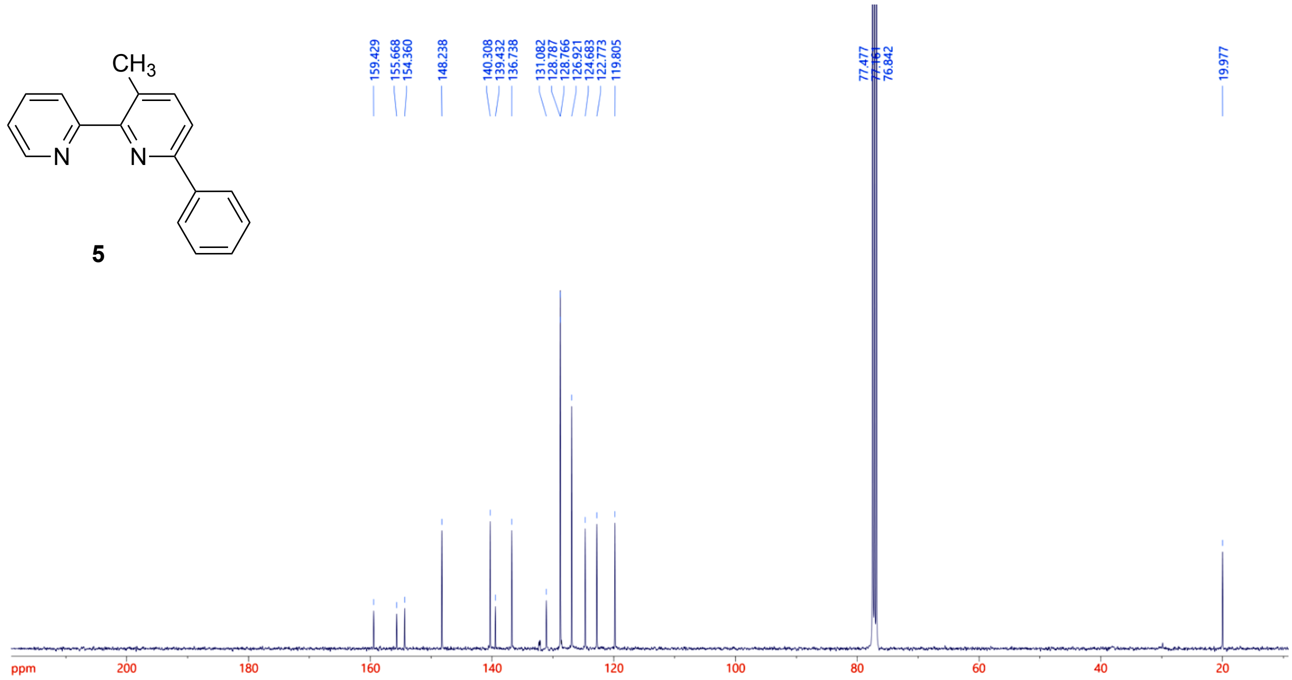

^{13}C NMR spectrum of compound **5** (aromatic region, $CDCl_3$)

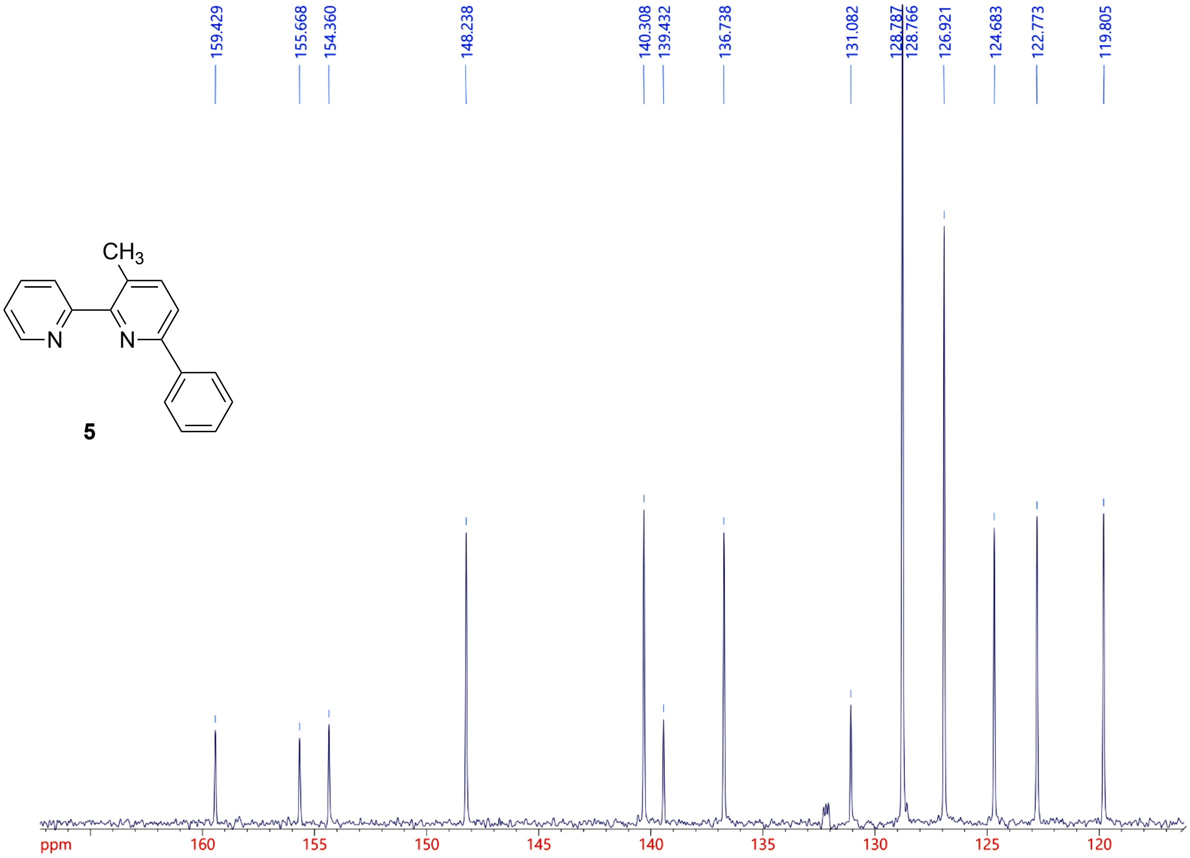

2D COSY NMR spectrum ($CDCl_3$) of compound **5**

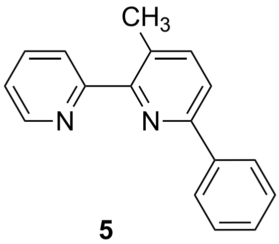

5

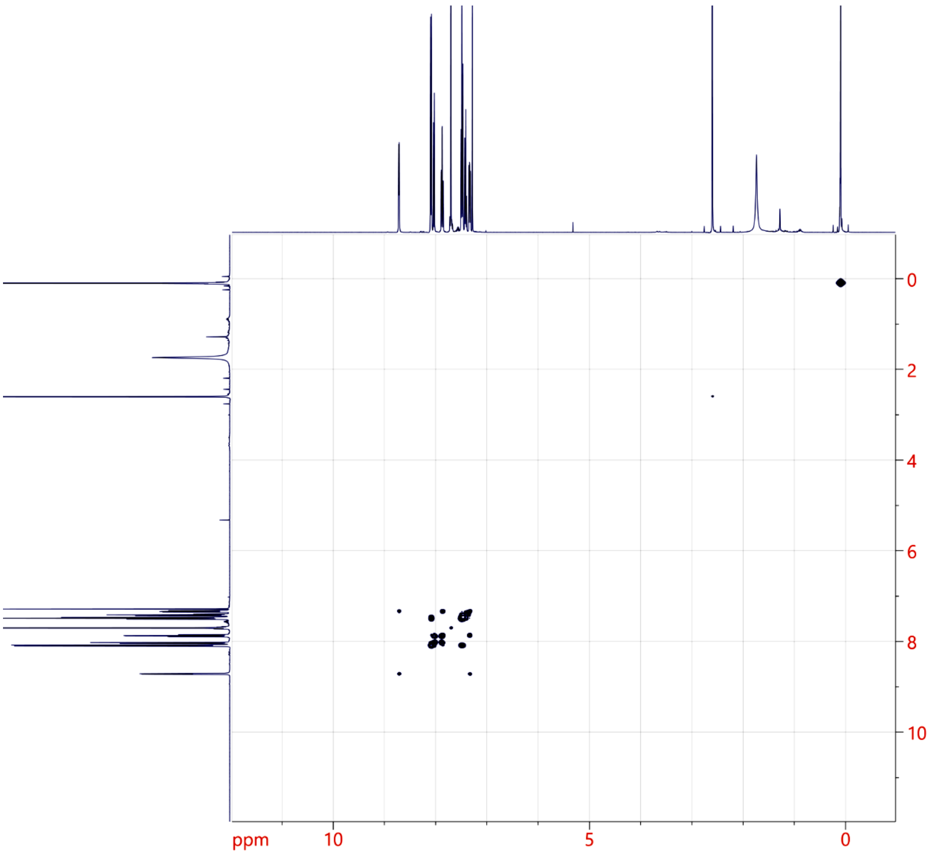

2D COSY NMR spectrum of compound **5** (aromatic region, $CDCl_3$)

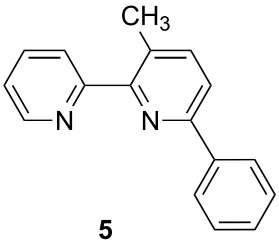

5

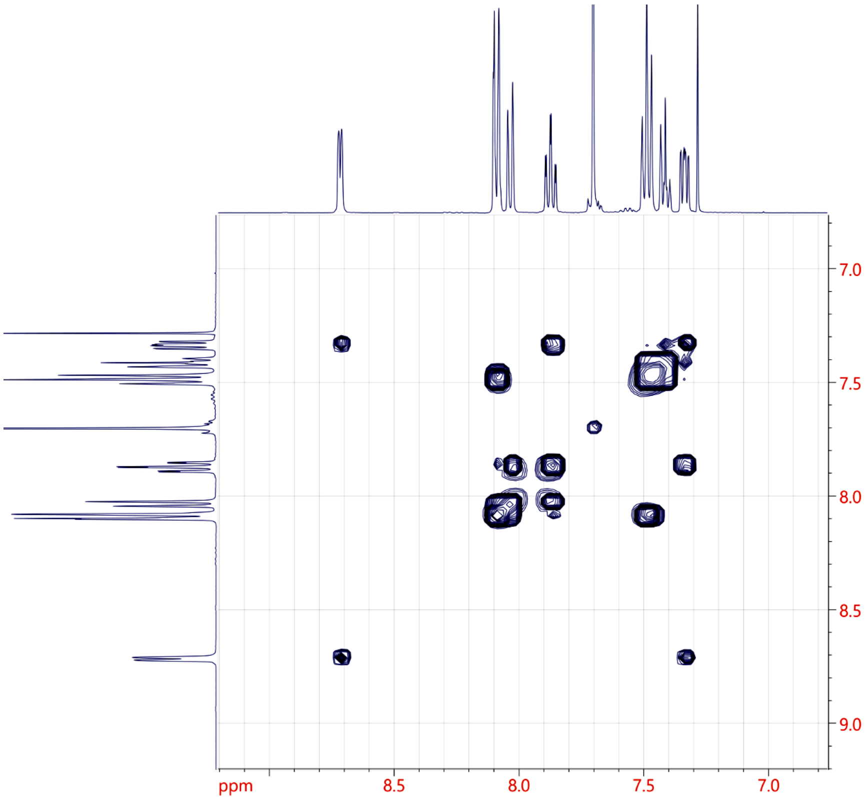

2D H-C HSQC NMR spectrum of compound **5** ($CDCl_3$)

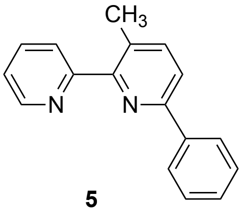

5

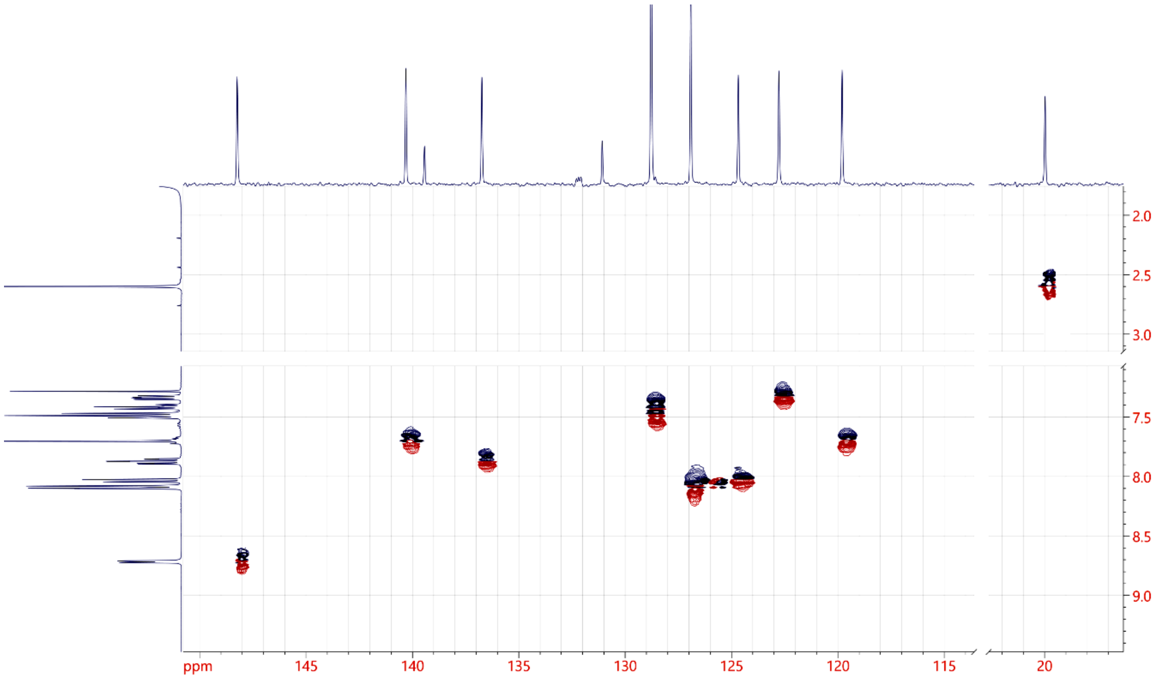

2D H-C HMBC NMR spectrum of compound **5** ($CDCl_3$)

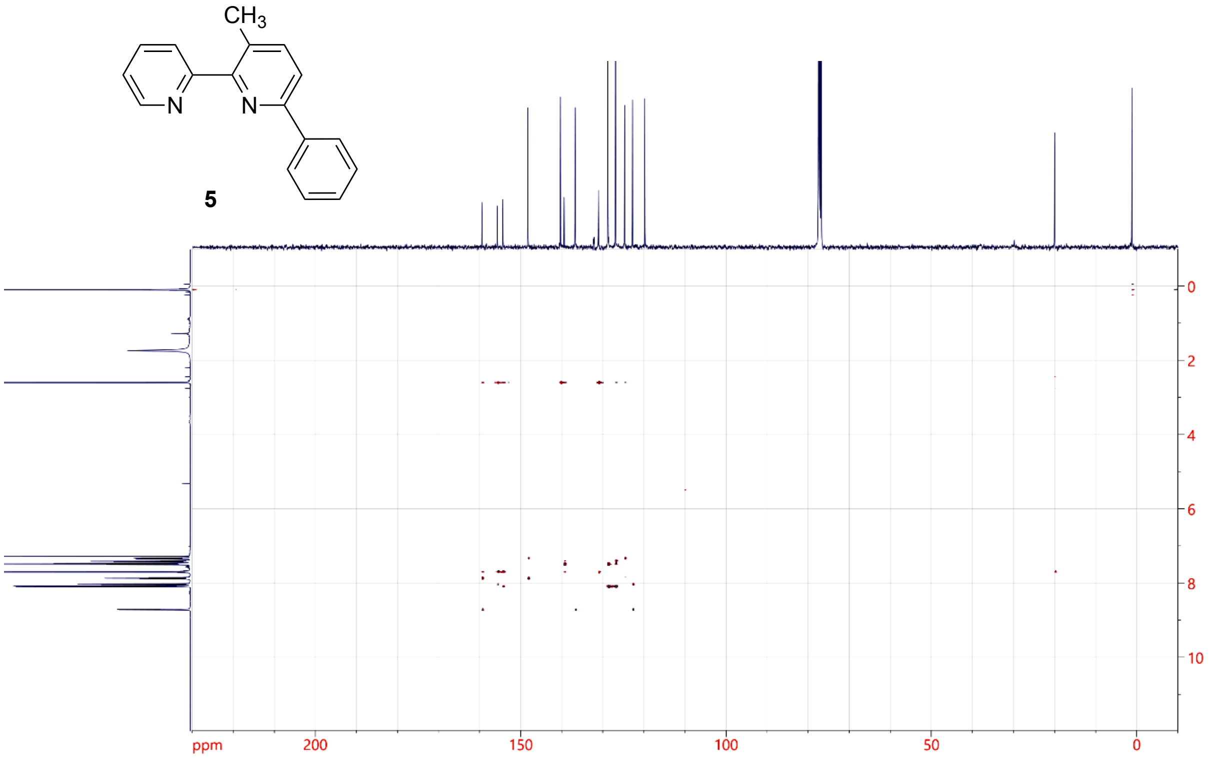

2D H-C HMBC NMR spectrum of compound **5** ($CDCl_3$, expanded)

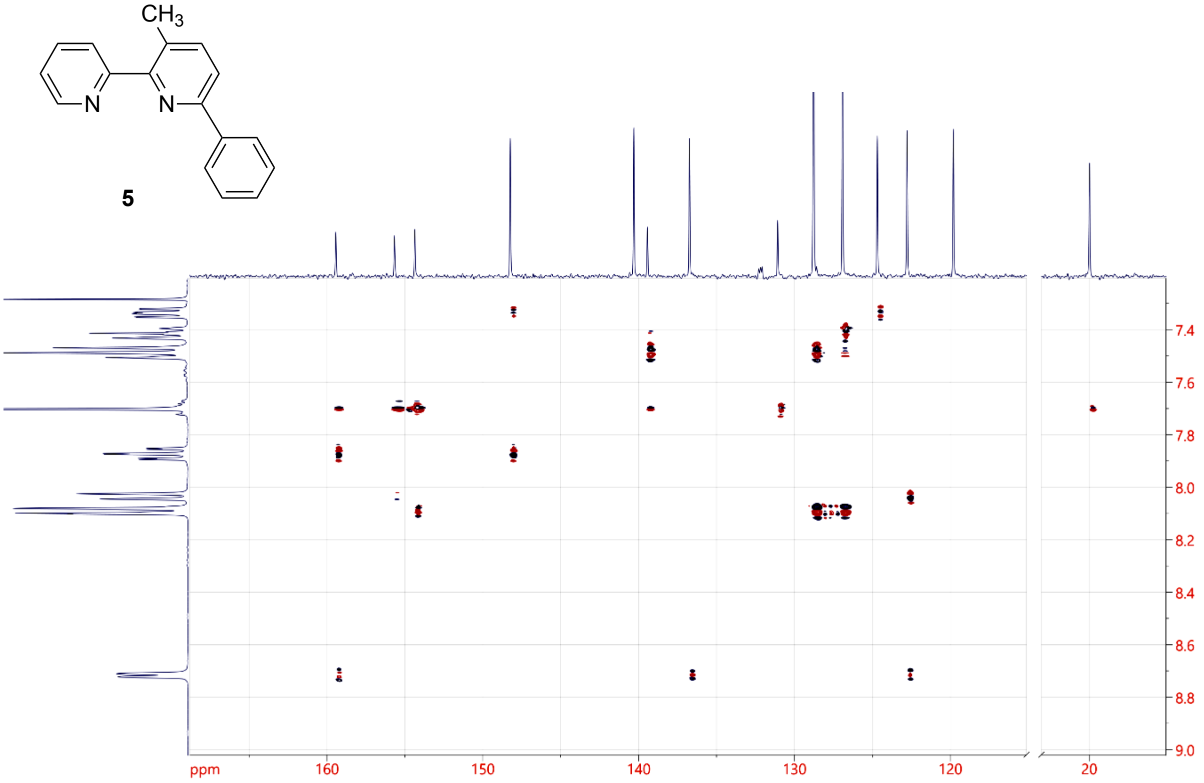

2D NOESY NMR spectrum of compound **5** (CD_2Cl_2)

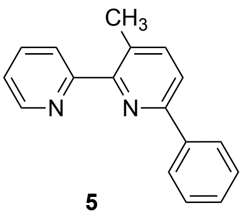

5

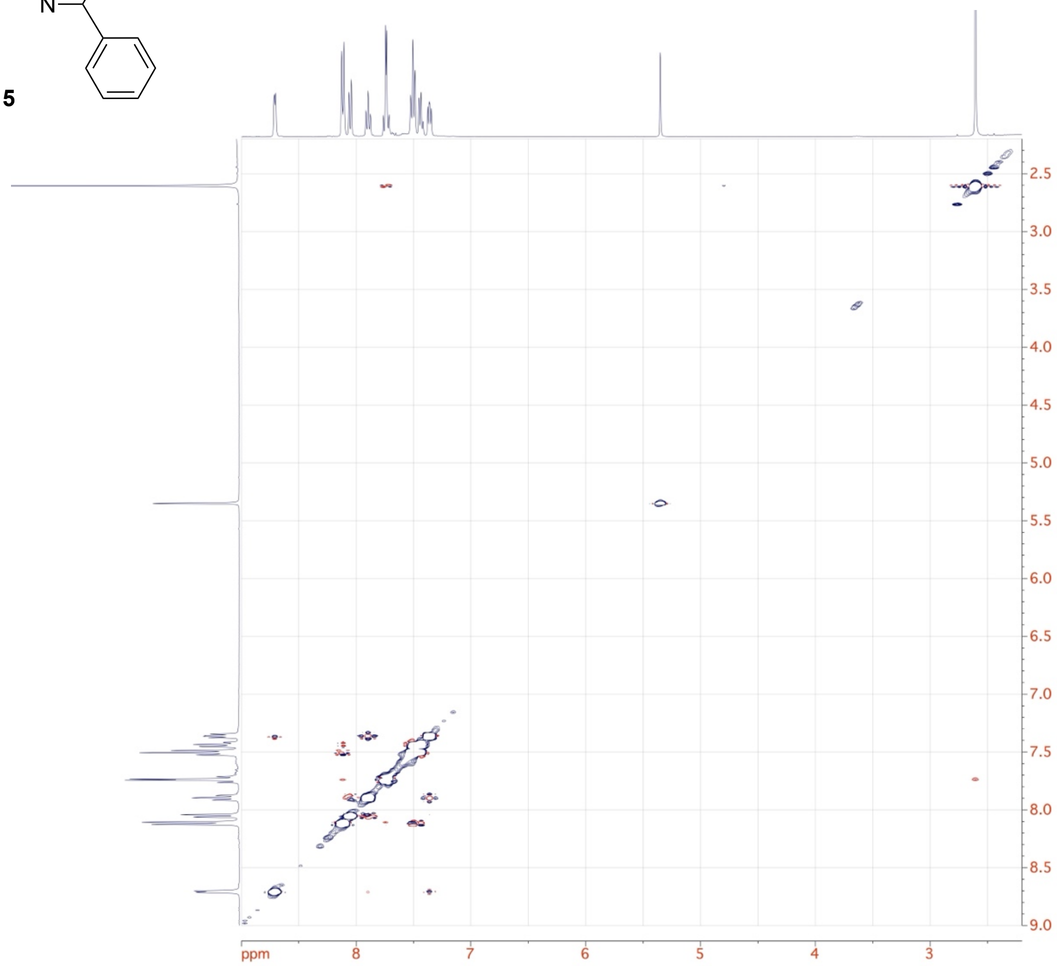

^{1}H NMR spectrum of complex **2a** ($CDCl_3$)

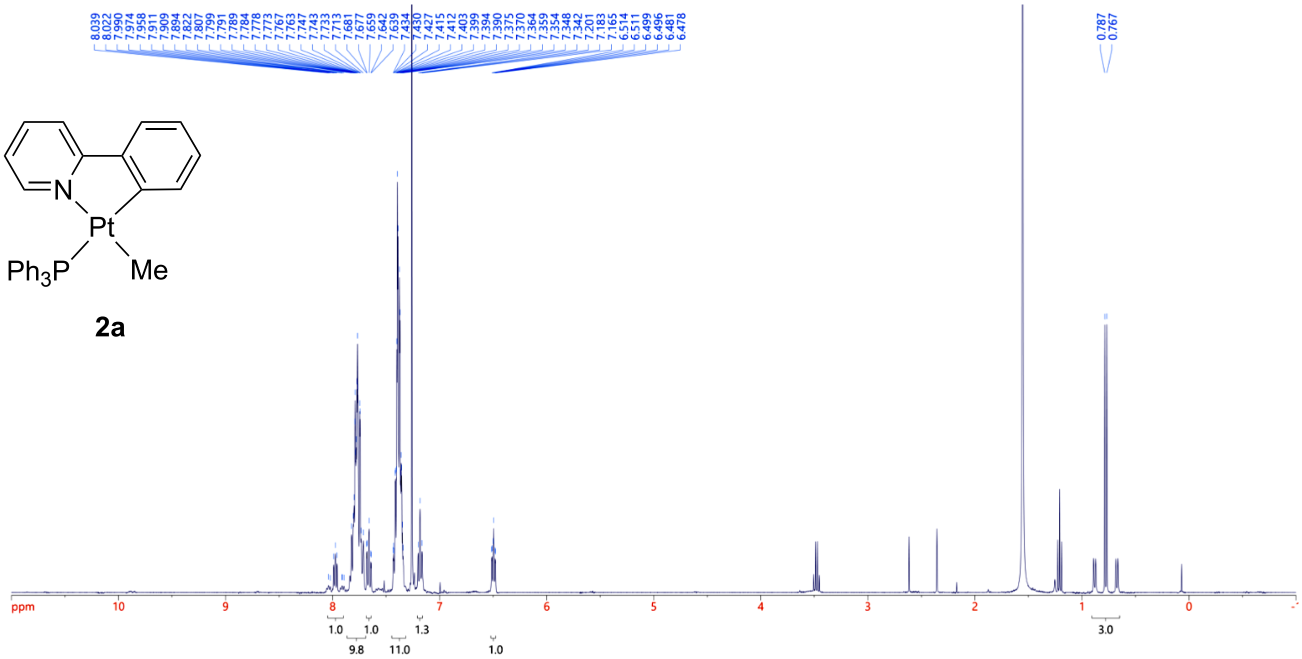

^{31}P NMR spectrum of complex **2a** ($CDCl_3$)

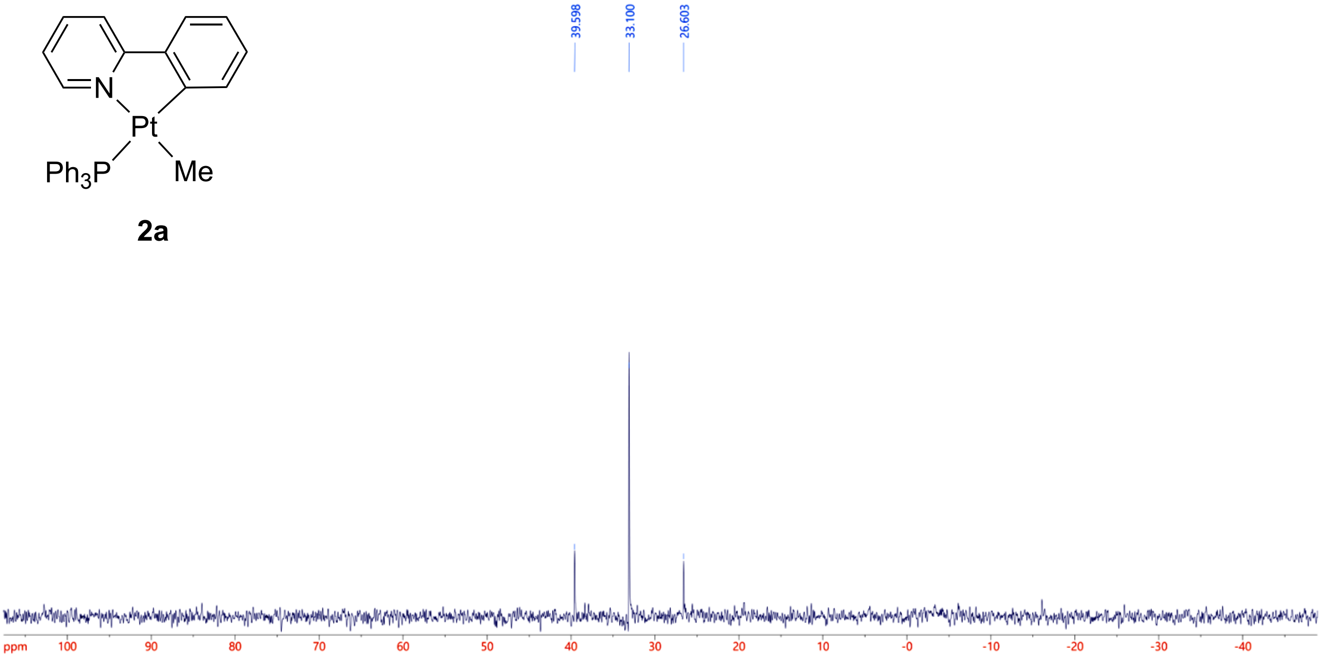

^{1}H NMR spectrum of complex **3a** ($CDCl_3$)

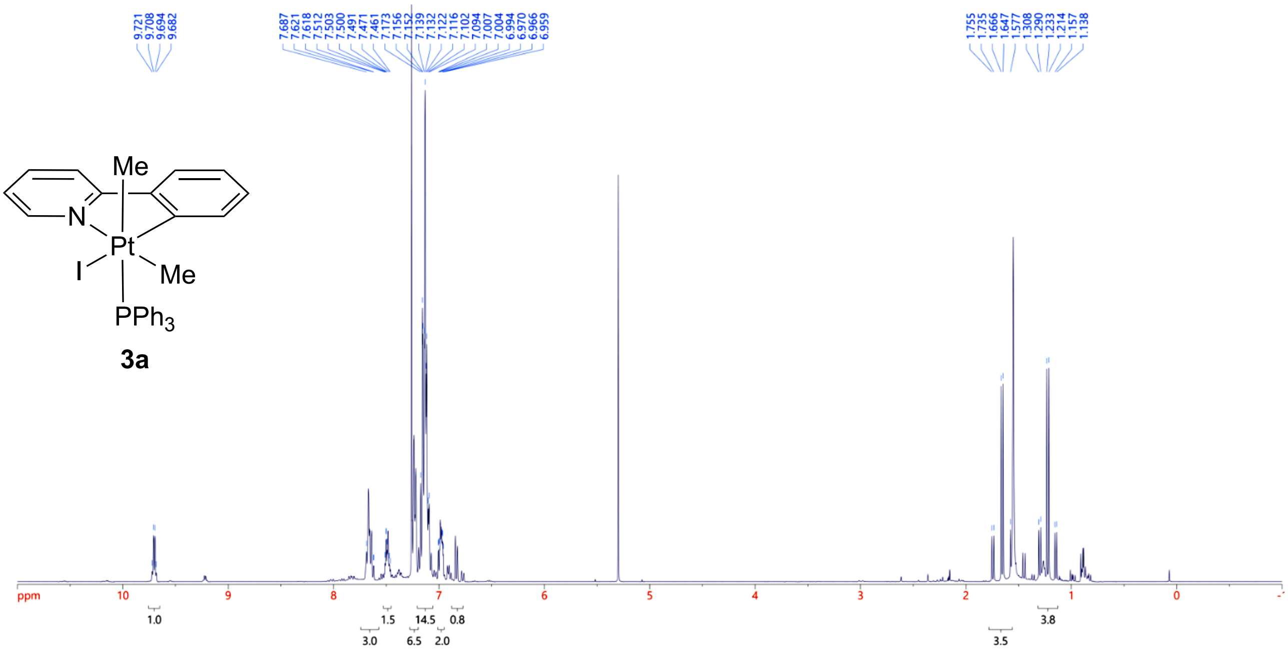

^{1}H NMR spectrum of complex **3a** ($CDCl_3$)

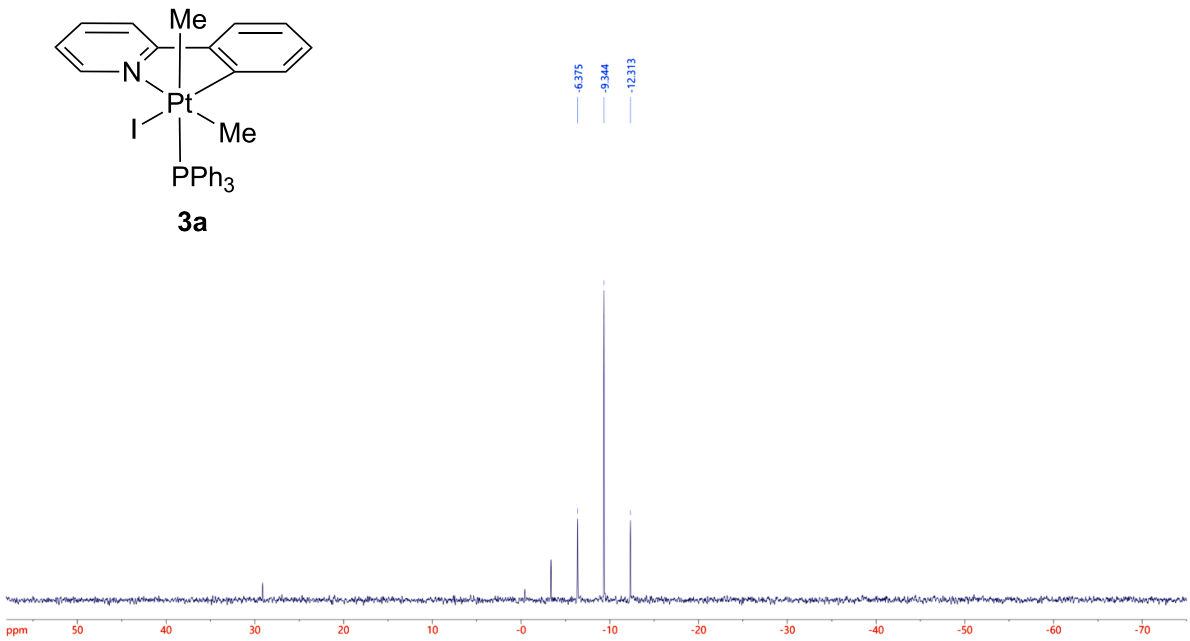

^{1}H NMR spectrum of compound **6** ($CDCl_3$)

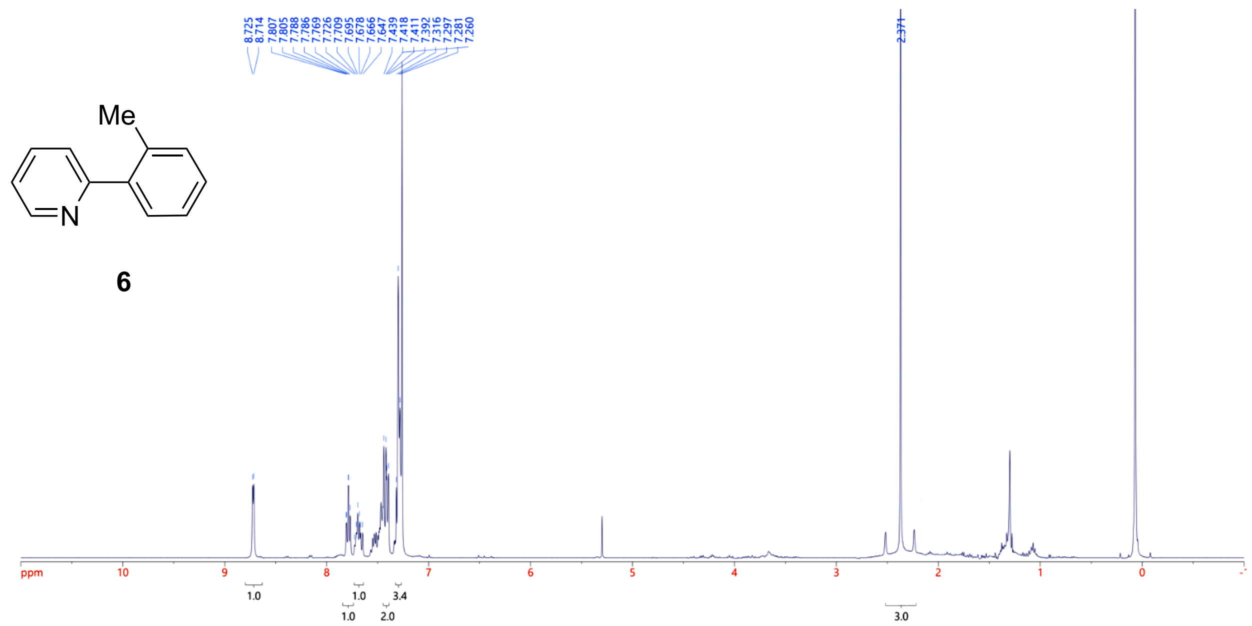

^{13}C NMR spectrum of compound **6** ($CDCl_3$)

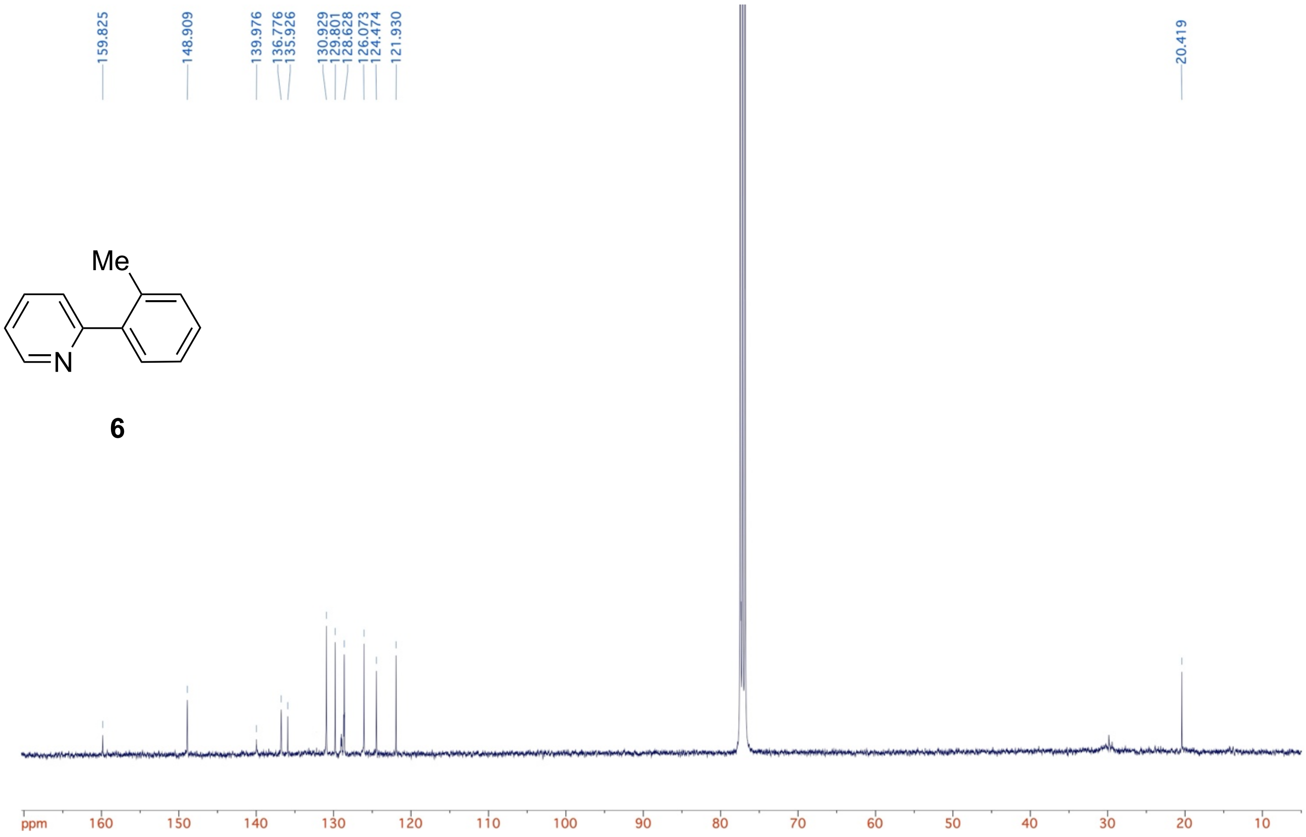

GC-MS spectrum of **5**

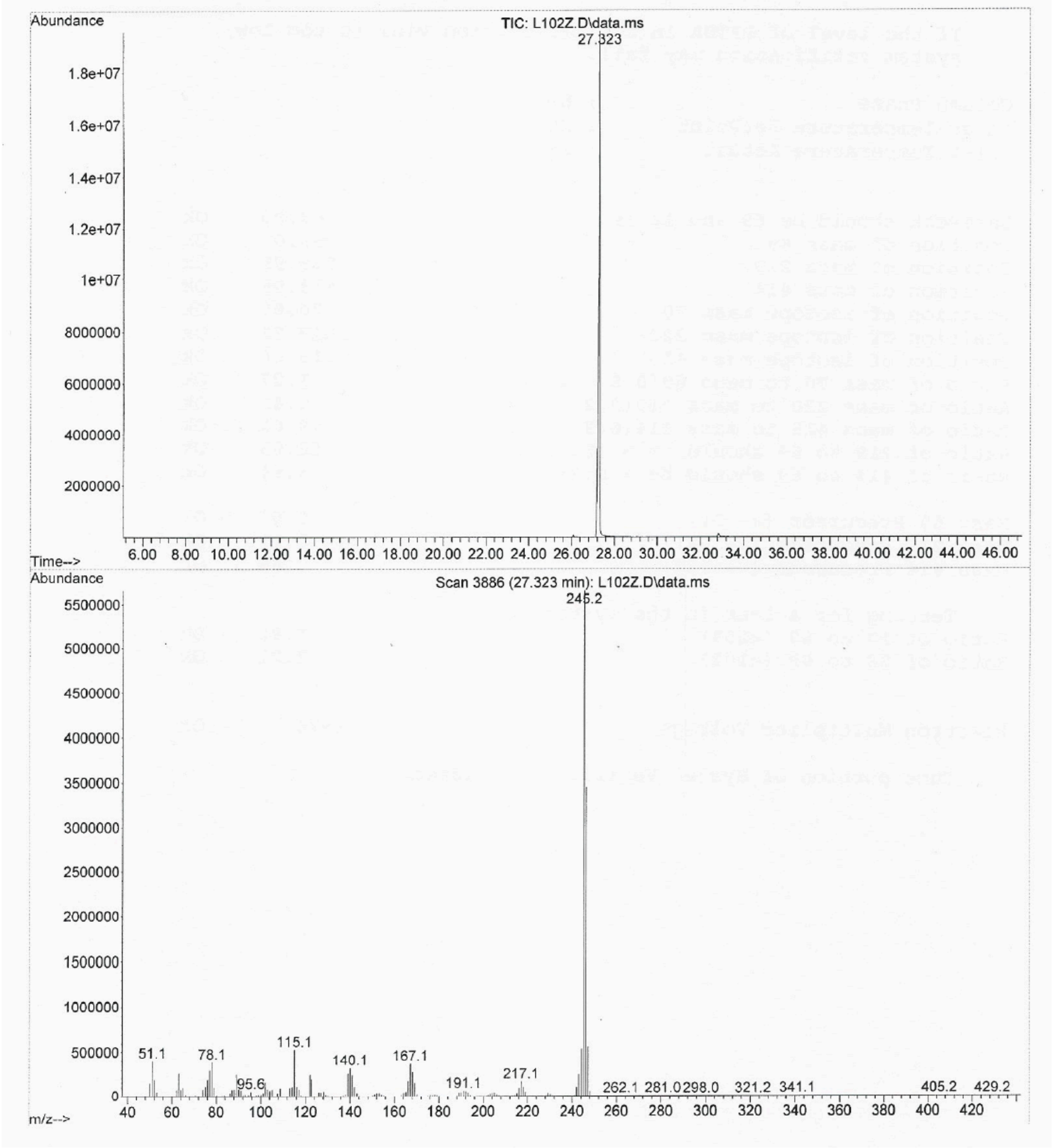

GC-MS spectrum of **6**

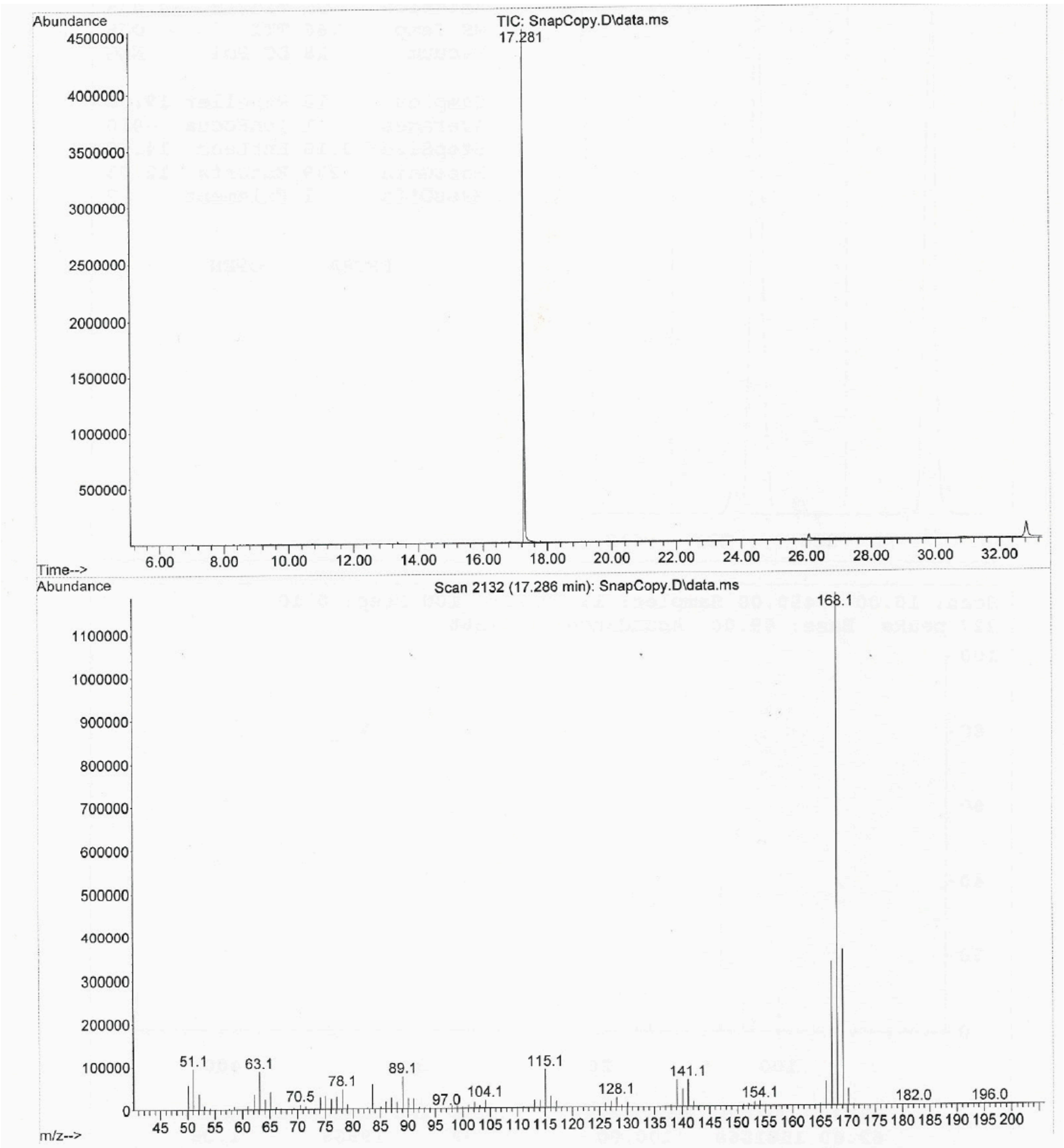

Supplement: Supplementary file 1 [file molecules-29-00707-s001.zip › molecules-2828104-supplementary.pdf]
